# PtrABF of *Poncirus trifoliata* functions in dehydration tolerance by reducing stomatal density and maintaining reactive oxygen species homeostasis

**DOI:** 10.1093/jxb/erv301

**Published:** 2015-06-25

**Authors:** Qinghua Zhang, Min Wang, Jianbing Hu, Wei Wang, Xingzheng Fu, Ji-Hong Liu

**Affiliations:** Key Laboratory of Horticultural Plant Biology (MOE), College of Horticulture and Forestry Science, Huazhong Agricultural University, Wuhan 430070, China

**Keywords:** ABRE, antioxidant enzyme, arginine decarboxylase, polyamine, polyamine oxidase, *Poncirus trifoliata*, ROS, stomatal development.

## Abstract

Abscisic acid-responsive element (ABRE)-binding factors (ABFs) play important roles in abiotic stress responses; however, the underlying mechanisms are poorly understood. In this study, it is reported that overexpression of *Poncirus trifoliata PtrABF* significantly enhanced dehydration tolerance. The transgenic lines displayed smaller stomatal apertures, reduced stomatal density/index, and lower expression levels of genes associated with stomatal development. PtrABF was found to interact with PtrICE1, a homologue of ICE1 (Inducer of CBF Expression 1) that has been shown to be critical for stomatal development. Microarray analysis revealed that a total of 70 genes were differentially expressed in the transgenic line, 42 induced and 28 repressed. At least two units of ABREs and coupling elements were present in the promoters of most of the induced genes, among which peroxidase and arginine decarboxylase were verified as bona fide targets of PtrABF. Transgenic plants exhibited higher antioxidant enzyme activities and free polyamine levels, but lower levels of reactive oxygen species (ROS) and malondialdehyde. Polyamines were revealed to be associated with ROS scavenging in the transgenic plants due to a modulation of antioxidant enzymes triggered by signalling mediated by H_2_O_2_ derived from polyamine oxidase (PAO)-mediated catabolism. Taken together, the results indicate that *PtrABF* functions positively in dehydration tolerance by limiting water loss through its influence on stomatal movement or formation and maintaining ROS homeostasis via modulation of antioxidant enzymes and polyamines through transcriptional regulation of relevant target genes.

## Highlight

PtrABF, a positive regulator of dehydration tolerance, is involved in stomatal development and regulates polyamine biosynthesis.

## Introduction

Plants are frequently challenged by a variety of environmental stresses, of which drought, salt, and cold are major impediments to the devastating impacts on plant growth and development and productivity. Since they are sessile, plants have evolved diverse and sophisticated defence mechanisms to tolerate or avoid such environmental challenges. One such adaptation involves the transcriptional reprogramming of numerous stress-responsive genes, whose products function, directly or indirectly, in preventing stress-derived damage (Shinozaki and Yamaguchi-Shinozaki, 2007; Shi *et al.*, 2015). Recent studies have revealed that many stress-responsive genes act in an abscisic acid (ABA)-dependent manner, implicating ABA as a major factor in the regulation of the complex molecular networks associated with abiotic stress responses (Fujita *et al.*, 2013; Mehrotra *et al.*, 2014). Exposure to abiotic stresses results in the accumulation of endogenous ABA, which in turn triggers the initiation of defence-related signal transduction pathways (Fujita *et al.*, 2013).

Significant progress has been made in identifying key signalling components involved in ABA-mediated gene regulation in response to unfavourable environmental stimuli, as represented by the exploitation of ABA receptors, pyrabactin resistance (PYR)/PYR1-like (PYL)/regulatory component of ABA receptors (RCAR) (Ma *et al.*, 2009; Park *et al.*, 2009). In the presence of ABA, the ABA receptors interact with and inhibit protein phosphatase 2C (PP2C), which serves as a negative regulator of ABA signalling. This leads to the activation of SnRK2 (sucrose non-fermenting 1-related protein kinase 2), which in turn phosphorylates transcription factors (TFs). The activated TFs can thus regulate an array of target genes by interacting with the relevant *cis*-acting elements in the corresponding promoters (Danquah *et al.*, 2014). Stress tolerance is largely dependent on both the number and induction intensity of the target genes at the bottom tier of the ABA signalosome. In this regard, the activated TFs play a critical and indispensable role in stress tolerance by relaying the upstream signals originating from ABA perception and modulating the downstream target genes. Plant genomes contain a large number of TFs involved in the regulation of the cascade of signalling events, including ABA-responsive element (ABRE)-binding factor (ABF) or AREB (ABRE-binding protein), which acts as master switches in the regulation of stress-responsive genes in the ABA-dependent signalling pathway (Kim, 2006; Fujita *et al.*, 2013; Liu *et al.*, 2014; Yoshida *et al.*, 2015).

ABFs/AREBs, members of the bZIP class of TFs, contain a highly conserved bZIP domain at their C-terminus that is composed of a basic region and a leucine repeat, as well as three additional conserved regions, designated as C1, C2, and C3, at the N terminus. The first characterized group of ABFs (ABF1, ABF2/AREB1, ABF3, and ABF4/AREB2) was isolated from *Arabidopsis thaliana* using a yeast one-hybrid (Y1H) assay (Choi *et al.*, 2000; Uno *et al.*, 2000; Kim *et al.*, 2004*b*). Since then, many stress-responsive ABF genes from other plant species have been reported, including tomato (Yáñez *et al.*, 2009; Hsieh *et al.*, 2010; Orellana *et al.*, 2010), rice (Hossain *et al.*, 2010), potato (Muñiz García *et al.*, 2012), and trifoliate orange (Huang *et al.*, 2010). The functions of several ABFs, and particularly those from *A. thaliana*, have been well characterized. Numerous studies have demonstrated that the overexpression of ABFs confers enhanced tolerance to single or multiple stresses (Kang *et al.*, 2002; Kim *et al.*, 2004*a*, *b*; Fujita *et al.*, 2005; Oh *et al.*, 2005; Vanjildorj *et al.*, 2005, 2006). These findings, together with the molecular characterization of *abf* mutants, have established ABFs as key regulators of abiotic stress responses and ABA signalling (Kim *et al.*, 2004a; Fujita *et al.*, 2005; Yoshida *et al.*, 2010, 2015).

ABFs regulate stress-responsive genes by binding to a specific *cis*-acting element in the promoter regions of target genes, known as the ABRE. Several studies comparing the large-scale transcriptomes between overexpressing lines or mutants and the wild type (WT) have identified sets of genes as potential ABF targets. For example, Oh *et al.* (2005) identified seven target genes of ABF3 using microarray analyses, while eight genes, including those encoding late embryogenesis abundant (LEA) class proteins and regulatory proteins, have been shown to be direct targets of ABF2 (Fujita *et al.*, 2005). In addition, Yoshida *et al.* (2010) identified several ABF target genes, including *LEA* genes, group-Ab *PP2C* genes, and TFs, through analysis of an *areb1 areb2 areb3* triple mutant. Due to the large number of ABA- and stress-responsive genes, it is clear that ABFs regulate a broad range of target genes, many of which are yet to be identified, and so current understanding of the involvement of ABFs in stress tolerance is substantially incomplete.

Previously a homologue of the *A. thaliana ABF4*, *PtrABF*, was isolated from trifoliate orange [*Poncirus trifoliata* (L.) Raf.], and it was demonstrated that its overexpression in tobacco led to enhanced drought tolerance (Huang *et al.*, 2010). However, molecular and physiological mechanisms underlying the observed stress tolerance provided by *PtrABF* were not fully investigated. In this study, it was therefore sought to identify the target genes of *PtrABF* and to investigate whether it has additional functions. The involvement of *PtrABF* in dehydration tolerance was characterized, and it was found that it influenced stomatal aperture and development. Furthermore, it was found that PtrABF physically interacts with PtrICE1, a homologue of ICE1 (Inducer of CBF Expression 1), which is known to control stomatal development (Kanaoka *et al.*, 2008). Microarray analysis revealed that expression levels of 70 genes associated with diverse aspects of stress responses were modified in the transgenic line. A large proportion of the induced genes contained ABREs and coupling elements (CEs) in their promoters, among which peroxidase (*POD*) and arginine decarboxylase (*ADC*) were confirmed as two bona fide targets of PtrABF. Meanwhile, the overexpressing lines exhibited higher antioxidant enzyme activities and increased free polyamine content, but had lower levels of reactive oxygen species (ROS) and malondialdehyde (MDA) compared with the WT. Interestingly, treatment of the transgenic plants with d-arginine resulted in elevated ROS levels, while guazatine treatment decreased H_2_O_2_ accumulation in response to dehydration. Taken together, the results indicate that overexpression of *PtrABF* altered stomatal movement and development, and alleviated ROS accumulation by modulating the activity of antioxidant enzymes and levels of free polyamines, factors that all probably contributed to the observed enhanced dehydration tolerance.

## Materials and methods

### Plant materials

To obtain *PtrABF*-overexpressing lines, seeds of trifoliate orange were surface-sterilized and sown in germination medium, and maintained at 25±2 °C in the dark for 30 d before being transferred to a 16h photoperiod for a further 7 d. Stem segments were used as explants for transformation of *PtrABF* (Huang *et al.*, 2010). Transformation, selection, and regeneration of trifoliate orange were carried out as previously described (Fu *et al.*, 2011). Transgenic plants containing the *NPTII* gene were used as a negative control. For further details of this and the following sections see the Supplementary methods available at *JXB* online.

### Molecular characterization of the regenerated plants

To confirm the presence of the transgene in the putative transgenic plants, genomic PCR analysis was performed using primers NPTII and PtrABF-1 (Supplemenetary Table S1 at *JXB* online). The transcript levels of *PtrABF* and *NPTII* in the positive lines were analysed by quantitative real-time PCR (qPCR) and reverse transcription–PCR (RT–PCR), respectively.

### Dehydration treatment of the transgenic lines

For the water loss assay, fully expanded leaves of plants growing under normal conditions were detached, and dehydrated for 90min. The leaves were collected before and/or after dehydration for physiological assays or molecular analysis. In addition, the transgenic lines and WT were pre-treated with d-arginine or guazatine before they were exposed to dehydration, followed by ROS detection or analysis of activity and expression levels of antioxidant enzymes. The dehydration treatment was repeated three times, giving consistent results. One repetition contained three replicates, which was composed of at least seven leaves.

### Analysis of electrolyte leakage, cell death, and MDA and ROS accumulation

Electrolyte leakage (EL) was measured as previously described (Shi *et al.*, 2010). Cell death of the leaves was examined by histochemical staining with trypan blue (Pogány *et al.*, 2009). ROS (O_2_^–^, H_2_O_2_) and MDA in the dehydrated leaves were histochemically detected or quantitatively measured. In addition, H_2_O_2_ in the guard cells was detected by staining assay with H2DCF-DA.

### Analysis of stomatal characteristics

Stomatal apertures were examined before and after dehydration using the abaxial epidermis of leaves detached from 6-week-old plants. The stomatal density (the number of stomata per unit of area), stomatal area, and stomatal index (number of stomata/total number of epidermal cells) were determined using leaves collected before the stress treatment. All of the examinations were done using ImageJ software.

### Measurement of free polyamines and antioxidant enzyme activities

Extraction of free polyamines was performed based on the method described by Liu and Moriguchi (2007). Free polyamines were derivatized and quantified by high-performance liquid chromatography (HPLC). Antioxidant enzymes were extracted and homogenized in 5ml of extraction buffer containing 50mM phosphate buffer (pH 7.8) and 1% polyvinylpyrrolidone. The enzyme activities were measured using relevant kits according to the manufacturer’s instructions.

### Microarray analysis

Leaves harvested from 60-day-old plants growing under normal conditions were used for microarray analysis. The microarray data were processed (Huang *et al.*, 2013) to identify differentially expressed genes (DEGs) in the transgenic line #10. The microarray results were validated by qPCR analysis.

### Yeast one-hybrid assay

The promoters of *POD* (Cit.17340.1.S1_at) and *ADC* (Cit.17713. 1.S1_s_at), designated as *PtrPOD* and *PtrADC*, respectively, were acquired and analysed to identify ABREs and CEs. An effector vector pGADT7-*PtrABF* and two reporter vectors, pAbAi-pPOD’ (a fragment of the *PtrPOD* promoter, 161bp) or pAbAi-pADC’ (a fragment of the *PtrADC* promoter, 214bp), were generated. The Y1H assay was carried out following the manufacturer’s instructions (Clontech, USA).

### Transient expression assay

The partial promoter fragments were cloned into the reporter vector pGreenII 0800-LUC, while the full-length open reading frame (ORF) of *PtrABF* was fused into the pGreen II 62-SK 0029 binary vector to generate an effector. Transient expression assays were carried out using tobacco protoplast transformation.

### Yeast two-hybrid assay

A bait vector pDEST32/PtrICE1 (with the GAL4 DNA-binding domain) and a prey vector pDEST22/PtrABF (with the GAL4 activation domain) were constructed The yeast two-hybrid assay was performed using the ProQuest™ Two-Hybrid System following the manufacturer’s protocol (Invitrogen, USA).

### Bimolecular fluorescence complementation (BiFC) and subcellular localization assays

The full-length coding sequence of *PtrABF* was cloned into pSPYCE-35S (PtrABF–cYFP), and PtrICE1 was introduced into pSPYNE-35S (PtrICE1–nYFP). *AtbZIP63* was used as a positive control, while PtrICE1–nYFP and cYFP were used as negative controls. *Agrobacterium tumefaciens*-mediated leaf infiltration was performed according to Walter *et al.* (2004) with appropriate modification, followed by microscopic observation. Subcellular localization of PtrABF and PtrICE1 was carried out using transient transformation of tobacco leaves.

## Statistical analysis

The data were statistically processed using the SAS statistical software package (SAS Institute); statistical difference was compared using ANOVA (analysis of variance) based on Fisher’s LSD test, taking *P<0.05, **P<0.01, and ***P<0.001 as significantly different.

## Results

### Overexpression of PtrABF in trifoliate orange confers enhanced dehydration tolerance

To verify the role of *PtrABF* in dehydration tolerance, trifoliate orange transgenic plants were generated via *Agrobacterium*-mediated transformation of epicotyls using a construct containing the transgene under the control of the *Cauliflower mosaic virus* (CaMV) *35S* promoter. Seven positive transgenic lines were confirmed by genomic PCR analysis (Supplementary Fig. S1a, b at *JXB* online), and qPCR analysis showed that the transgene was highly overexpressed in two of these lines (#8 and #10) when compared with the WT (Supplementary Fig. S1c). The transgenic plants and WT were indistinguishable in plant morphology under normal growth conditions.

**Fig. 1.**
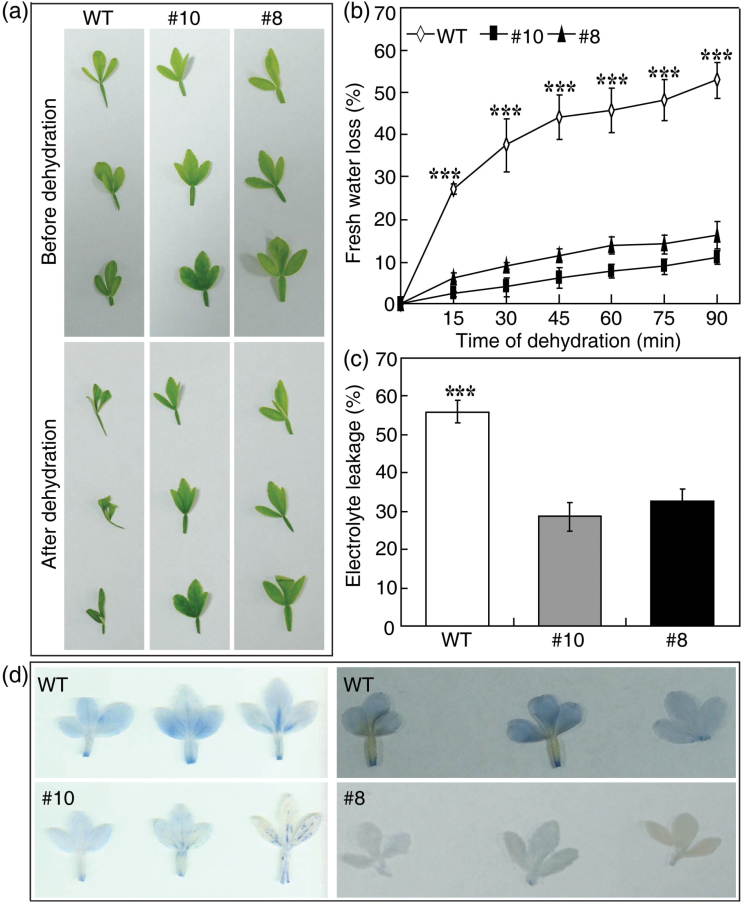
Overexpression of *PtrABF* in trifoliate orange confers enhanced dehydration tolerance. (a) Phenotypes of leaves detached from 60-day-old transgenic lines (#8 and #10) and the wild type (WT) before and after 90min of dehydration treatment under an ambient environment. (b) Rates of water loss from the detached leaves of the WT and transgenic lines during the dehydration treatment, measured every 15min. Error bars indicate the SD (*n*=3). (c, d) Electrolyte leakage (c) and cell death (d) in the WT and transgenic lines measured after dehydration stress. Error bars indicate the SD (*n*=3). Asterisks indicate a significant difference between the WT and transgenic lines (***P<0.001). (This figure is available in colour at *JXB* online.)

To assess the dehydration tolerance, fully expanded leaves detached from 60-day-old WT and transgenic plants were dehydrated for up to 90min on filter paper under ambient conditions. As seen in Fig. 1a, the treatment resulted in more severe wilting and rolling of WT leaves than those from the transgenic lines. The fresh weight (FW) of the leaves was measured every 15min to evaluate the rate of water loss. Consistent with the phenotype test, the WT leaves lost more water than those from the two transgenic lines during dehydration. In particular, the most significant difference in amount and rate of water loss between the WT and the transgenic lines was detected within 15min of dehydration. At the end of the dehydration treatment, the WT leaves exhibited a water loss of 55.6%, whereas the transgenic leaves lost only 10.9–16.1% of their water (Fig. 1b). EL, a parameter indicating the degree of membrane damage (Huang *et al.*, 2013), was also evaluated in parallel. After 90min of dehydration, EL in the WT (55.9%) was nearly twice that of the two transgenic lines (28.6% for #10, 32.5% for #8) (Fig. 1c). Trypan blue staining, a sensitive assay for monitoring cell death (Pogány *et al.*, 2009), was performed on both WT and transgenic lines after the dehydration. The WT leaves were found to stain much more strongly than leaves from the transgenic lines (Fig. 1d), indicating more cell death in WT leaves. Thus, overexpression of *PtrABF* in trifoliate orange led to enhanced dehydration tolerance.

As a further control, the dehydration tolerance of WT and transgenic plants containing the neomycin phosphotransferase II gene (*NPTII*) was also evaluated. Integration and overexpression of the *NPTII* gene in the transgenic plants were confirmed by genomic PCR and RT–PCR, respectively (Supplementary Fig. S2a, b at *JXB* online). The WT and negative control plants displayed similar leaf morphology after exposure to dehydration for 90min (Supplementary Fig. S2c). The water loss rate and cell death staining profiles were also indistinguishable between WT and transgenic lines (Supplementary Fig. S2d, e). These data indicate that the presence of the *NPTII* gene did not alter the dehydration tolerance of the transgenic plants, thus confirming that the improved tolerance of the *PtrABF*-overexpressing plants was conferred by *PtrABF*.

### Transgenic plants have different stomatal apertures, density, and index

**Fig. 2.**
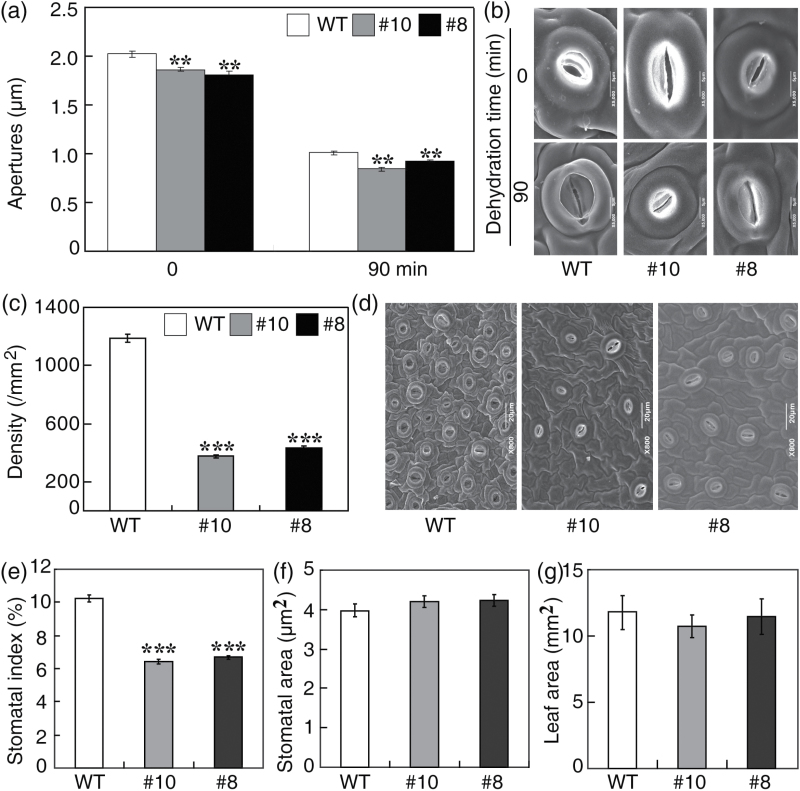
Comparison of stomatal parameters between the wild-type (WT) and transgenic lines. (a) Stomatal aperture size of WT and transgenic lines, measured in fully expanded leaves before and after 90min of dehydration. Error bars indicate the SD (*n*≥30). (b) Scanning electron microscopy (SEM) images showing representative stomata of the WT and transgenic lines before (upper panel) and after (bottom panel) 90min of dehydration. Scale bars=5 μm. (c) Stomatal density in fully expanded leaves collected from WT and transgenic plants grown under normal conditions. Error bars indicate the SD (*n*=22). (d) Representative SEM photographs showing the difference in the number of stomata between the WT and transgenic lines. Scale bars=5 μm. (e–g) Stomatal index (e), stomatal area (f), and leaf areas (f) of the WT and transgenic lines. Asterisks indicate significant differences between the transgenic lines and the WT at the same time point (**P<0.01; ***P<0.001).

The fact that the transgenic lines overexpressing *PtrABF* lost less water than WT plants prompted the investigation of whether there was a difference in leaf stomatal apertures, an important determinant of leaf water loss. Without dehydration treatment, the transgenic lines exhibited smaller leaf stomatal openings compared with the WT. Although dehydration treatment led to a noticeable decrease in the stomatal apertures of all tested lines, apertures of the transgenic plants were still smaller than those of the WT (Fig. 2a, b). However, it has to be pointed out that the difference in stomatal apertures between the transgenic lines and the WT is not substantial. In contrast, the transgenic lines and WT displayed much greater gaps in the stomatal density of the leaf epidermis; the mean stomatal density of WT leaves was 1,186mm^–2^, while that of leaves from #10 and #8 ranged from 375mm^–2^ to 431mm^–2^ (Fig. 2c, d). Thus, overexpression of *PtrABF* led to a prominent reduction in stomatal density, which is consistent with the lower water loss rates observed in the transgenic lines. Stomatal indices of the transgenic lines were significantly lower than that of the WT (Fig. 2e). However, when stomatal area and leaf areas of the tested lines were examined, no remarkable differences were detected (Fig. 2f, g).

**Fig. 3.**
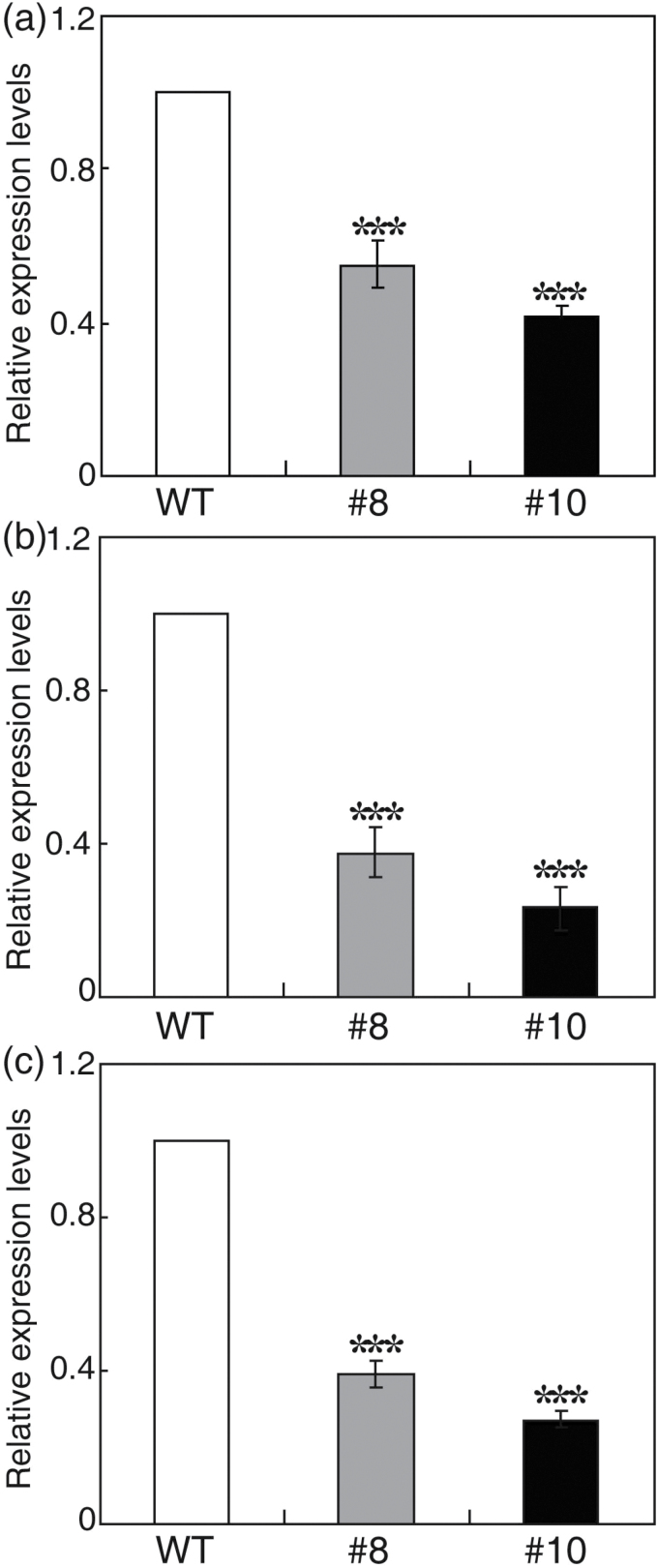
Expression profiles of genes associated with stomatal development, using fully expanded leaves sampled at the same stage as experimental tissues. (a–c) Expression patterns of *SPCH* (a), *FAMA* (b), and *MUTE* (c) in the transgenic lines (#8 and #10) and wild type (WT) based on qPCR analysis. Error bars indicate the SD (*n*=4). Asterisks indicate significant differences between the transgenic lines and the WT (**P<0.01, ***P<0.001).

Three closely related basic helix–loop–helix (bHLH) genes, *SPEECHLESS* (*SPCH*), *MUTE*, and *FAMA*, have been reported to act as key regulators of stomatal development (Kanaoka *et al.*, 2008). It was therefore investigated whether the altered stomatal phenotypes of the transgenic plants correlate with differences in expression of these genes. The transcript levels of the homologous genes from trifoliate orange (Supplementary Table S2 at *JXB* online) were examined by qPCR. As shown in Fig. 3, steady-state mRNA levels of the three genes were shown to be substantially lower in the two transgenic lines relative to the WT.

### PtrABF physically interacted with PtrICE1

**Fig. 4.**
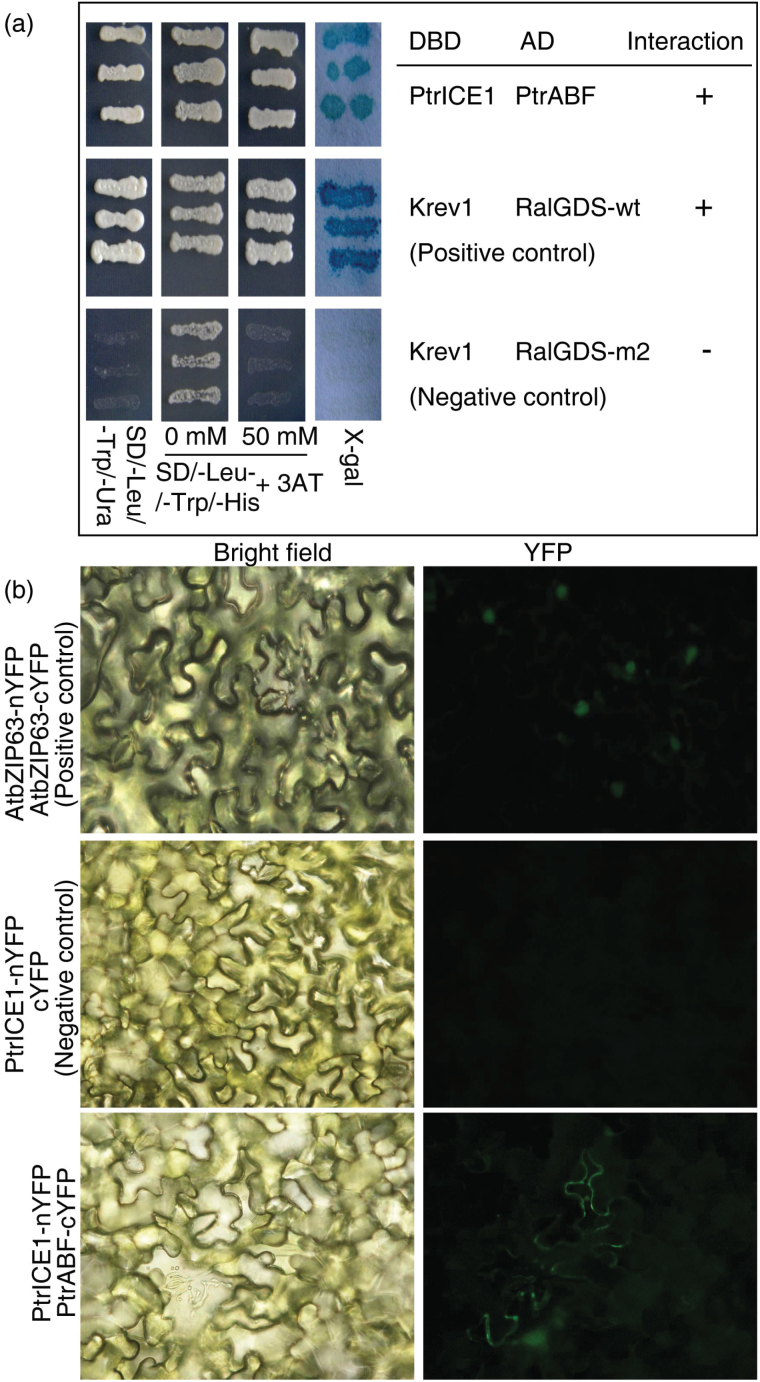
Analysis of the interaction between PtrABF and PtrICE1 by yeast two-hybrid assay and bimolecular fluorescence complementation (BiFC). (a) Yeast two-hybrid analysis of the interaction between PtrABF and PtrICE1. Growth of the yeast cells on SD/-Leu/-Trp/-Ura or SD/-Leu/-Trp/-His with or without added 3-AT. The blue colour shows the examination of X-gal activity of the corresponding yeast cells. (b) BiFC assay of the interaction between PtrABF and PtrICE1 using tobacco leaf epidermis. Images taken under bright field and fluorescence are shown. Positive and negative controls are used to verify the reliability of the approach.

Recently, the *A. thaliana ICE1* gene has been shown to interact with the above-mentioned bHLH genes, thus acting as the central component of the core regulatory unit of stomatal differentiation (Kanaoka *et al.*, 2008). Since *PtrABF* overexpression reduced stomatal density in the transgenic lines, it was hypothesized that this might involve the interplay between PtrABF and PtrICE1, the *P. trifoliata* homologue of ICE1. First the subcellular localization of PtrABF and PtrICE1 was analysed using tobacco leaves, which showed that both of them are localized to the nuclei (Supplementary Fig. S3 at *JXB* online). Then a yeast two-hybrid assay was performed, using PtrABF as prey and PtrICE1 as bait, to test the interaction of these two proteins. The prey and bait vectors were co-transformed into yeast cells, which were cultured on SD/-Leu/-Trp/-Ura or SD/-Leu/-Trp/-His with or without 3-AT (3-amino-1, 2, 4-triazole). All of the yeast cells grew on SD/-Leu/-Trp/-His, but only the positive control and the PtrABF–PtrICE1 co-transformants survived on SD/-Leu/-Trp/-Ura and SD/-Leu/-Trp/-His with the 3-AT supplement. This result was further supported by an X-gal assay, showing interaction between PtrABF and PtrICE1 (Fig. 4a). To verify the interaction between PtrABF and PtrICE1 *in planta*, a BiFC assay was performed. PtrABF was fused to the C-terminal region of yellow fluorescence protein (YFP) to obtain the PtrABF–cYFP fusion protein, while PtrICE1 was fused to the N-terminal region of YFP to generate PtrICE1–nYFP. Co-transformation of PtrABF–cYFP and PtrICE1–nYFP resulted in fluorescent signals in the cytoplasm of tobacco (*Nicotiana benthamiana*) leaf epidermal cells (Fig. 4b), confirming a physical interaction between PtrABF and PtrICE1.

### The transcriptome of PtrABF-overexpressing lines

**Fig. 5.**
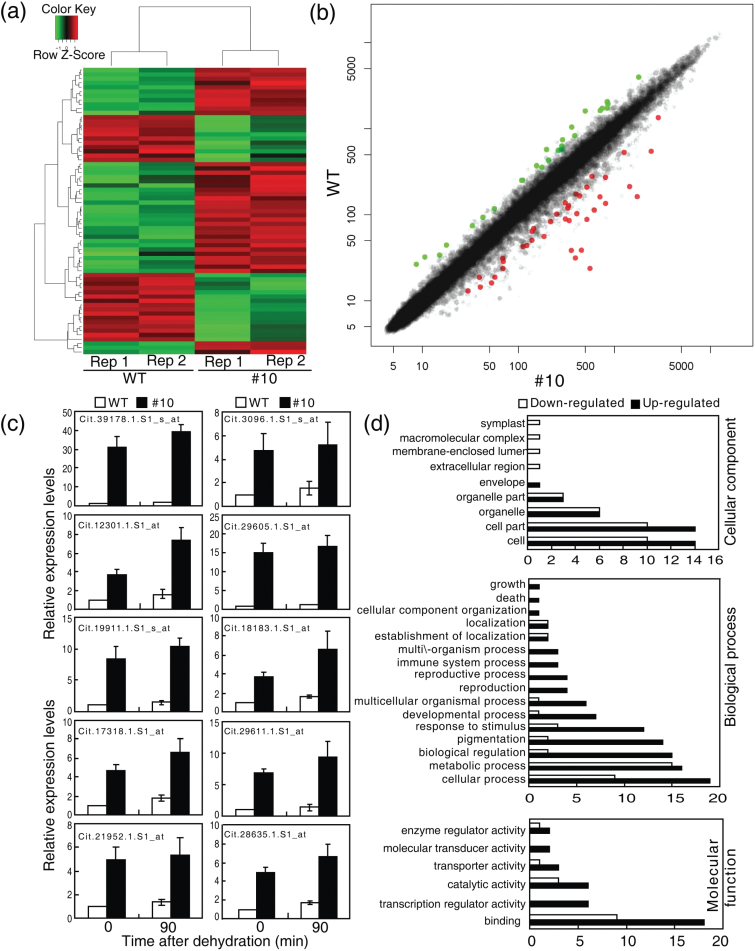
Differentially expressed genes (DEGs) in the *PtrABF*-overexpressing line (#10). (a) Expression patterns of the DEGs in #10 relative to the wild type (WT). (b) A scatterplot of the expression profiles of the complete gene set in the *PtrABF*-overexpressing line relative to the WT. The red and green dots indicate the probe sets with a signal ratio >2 or <0.05, respectively, between #10 and the WT. (c) Expression patterns of 10 DEGs in the WT and #10 before and after dehydration treatment. Transcript levels of the genes in the sampled leaves were examined by qPCR. Error bars indicate the SD (*n*=4). (d) GO analysis of the DEGs based on cellular component, biological process, and molecular function.

To gain insights into the molecular mechanisms underlying the enhanced dehydration tolerance of the transgenic lines and to identify the potential downstream target genes of PtrABF, a genome-wide microarray analysis was performed using the Affymetrix Genechip Citrus Genome Array. RNA samples used as probes for hybridization with the chips were isolated from uniform leaves collected from the WT and transgenic line #10 grown under normal conditions. A total of 70 genes displayed a ≥2.0-fold difference in expression levels (*P*≤0.05) in the transgenic line when compared with the WT, with 42 and 28 genes showing a higher or lower expression, respectively, in the former (Fig. 5a, b; Supplementary Table S3 at *JXB* online). Relative expression levels of 10 of the genes that were induced by the microarray data were also quantified by qPCR analysis. The expression patterns indicated by the two approaches were similar, thus validating the reliability of the microarray results. The dehydration treatment induced the expression of the tested genes in both the WT and #10, but higher transcript levels were observed in the latter (Fig. 5c). Gene Ontology (GO) analysis of the DEGs revealed a distribution among various categories, of which ‘cellular process’, ‘binding’, and ‘cell’ were represented most extensively in ‘biological process’, ‘molecular function’, and ‘cellular component’, respectively (Fig. 5d). Of the 42 induced DEGs, 33 were functionally annotated (Table 1). Nine encode TFs of the NAC, WRKY, and bZIP families; seven played a role in mediating stress-associated signal transduction. Ten genes encode proteins associated with oxidative stress, redox modulation, and responses to stimulus and stress, including peroxidase, LEA4-5, and dehydration-induced protein. In addition, five genes are implicated in the metabolism of lipids, polyamines, and glycerol, while two genes are involved in development and transport.

**Table 1.** *A list of 33 genes induced in the transgenic line (#10) overexpressing* PtrABF *in comparison with the wild type, as revealed by microarray analysis, and their promoter information* The listed genes that can be functionally annotated, with a fold change ≥2.0 (*P*≤0.05), are included.

Biology function	Probe set ID	Product description	Fold change	Promoters		
				Length (bp)	No. of ABREs^*a*^	No. of CEs
Signal transduction	Cit.6937.1.S1_at	Calmodulin binding protein-like	10.6584	1631	0	0
	Cit.28233.1.S1	Calmodulin-binding protein	9.24319	2000	1	0
	Cit.19911.1.S1_s_at	CML23	5.98814	2058	2	2
	Cit.16885.1.S1_at	CML23	5.45979	2058	2	2
	Cit.21952.1.S1_at	CES101	2.90803	1766	0	0
	Cit.18183.1.S1_at	Calmodulin-binding receptor-like cytoplasmic kinase 2, CRCK2	2.6287	2664	2	0
	Cit.15386.1.S1_at	Signalling in sugar and nutrient physiology, GLR2.7	2.46976	2000	1	0
Transcription factors	Cit.12301.1.S1_at	bZIP60	2.38001	1504	2	0
	Cit.22360.1.S1_at	ANAC068	2.5932	2000	1	0
	Cit.29605.1.S1_at	NAC090	12.457	2029	3	1
	Cit.29611.1.S1_at	WRKY46	5.85275	2000	1	0
	Cit.32848.1.S1_at	ANAC087	2.43082	2075	0	0
	Cit.36234.1.S1_s_at	bZIP60	2.84623	2058	0	0
	Cit.3757.1.S1_at	WRKY46	4.42468	2566	1	1
	Cit.37829.1.S1_s_at	NAC090	11.7199	2029	3	1
	Cit.39642.1.S1_at	WRKY50	3.45106	2180	1	4
Oxidative stress and redox regulation	Cit.17340.1.S1_at	Peroxidases	2.91959	2000	1	2
	Cit.22418.1.S1_s_at	Redox thioredoxin	2.39812	2132	1	1
	Cit.25134.1.S1_at	Respiratory burst oxidase	2.22697	1328	1	1
	Cit.31330.1.S1_at	Respiratory burst oxidase	2.97614	2000	2	1
Response to stimulus and stress	Cit.39178.1.S1_s_at	LEA4-5	23.4362	1578	2	1
	Cit.17622.1.S1_at	Cold stress protein	2.13017	2000	2	1
	Cit.14915.1.S1_at	Dehydration-induced protein RD22-like protein 2	2.14237	2023	0	0
	Cit.22001.1.S1_at	Disease resistance protein (TIR-NBS-LRR class)	2.43452	2000	2	1
	Cit.24055.1.S1_at	PR-proteins	2.23615	878	1	1
	Cit.28635.1.S1_at	Disease resistance protein (TIR-NBS-LRR class)	3.95718	2000	2	0
Metabolic process	Cit.4457.1.S1_s_at	Lipid metabolism	6.76488	1988	2	1
	Cit.17318.1.S1_at	RING/U-box superfamily protein	3.31257	1183	2	1
	Cit.3096.1.S1_s_at	GK-2	2.97569	2714	1	1
	Cit.14586.1.S1_at	2-Oxoglutarate and Fe(II)-dependent oxygenase superfamily protein	2.45955	2000	3	0
	Cit.17713.1.S1_s_at	Arginine decarboxylase	2.17115	2503	1	3
Development	Cit.22036.1.S1_at	Development unspecified	2.78254	2000	1	1
Transport	Cit.39679.1.S1_s_at	UDP-galactose transporter 2,UTR2	2.29242	2046	0	1

^*a*^ The number of ABREs (ABA-responsive elements) and CEs (coupling elements) was examined using the promoter region of the relevant genes based on the citrus genome sequence data.

The promoters of the 33 induced and annotated DEGs were screened for the presence of ABREs and CEs based on the released citrus genome sequence (Table 1). The majority contained ABREs and CEs alone, or in combination, although five (Cit.6937.1.S1_at, Cit.21952.1.S1_at, Cit.32848.1.S1_at, Cit.36234.1.S1_s_at, and Cit.14915.1.S1_at) do not. The distribution of ABREs and CEs in the promoters could be classified into three types: 14 genes have at least two ABREs, with or without CEs in their promoters (Type I); nine carry one copy of ABRE and at least one CE (Type II), while the remaining five genes harbour only one ABRE or CE (Type III).

### PtrABF interacts with the promoters of PtrPOD and PtrADC

**Fig. 6.**
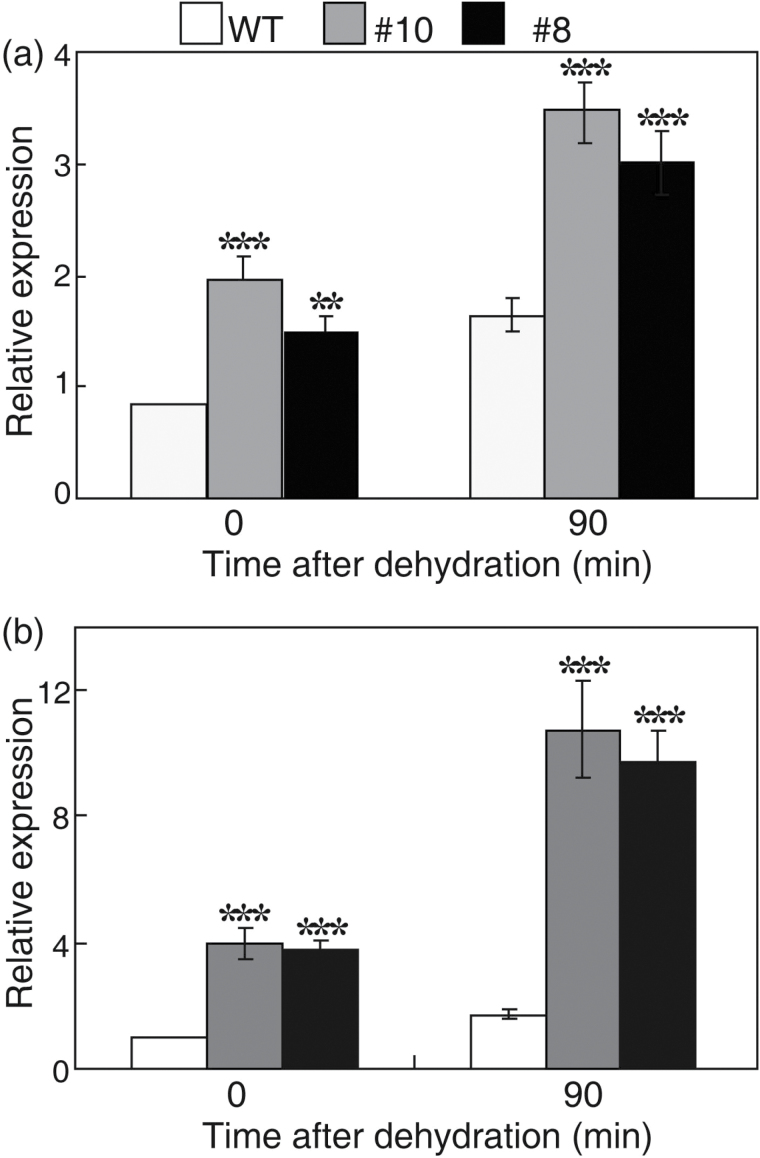
Overexpression of *PtrABF* alters the expression of *PtrPOD* and *PtrADC*. (a, b) Expression patterns of *PtrPOD* (a) and *PtrADC* (b) in the wild type (WT) and transgenic lines before and after dehydration treatment. Transcript levels of the tested genes were determined by qPCR. Error bars indicate the SD (*n*=4). Asterisks indicate significant differences between the transgenic lines and the WT at the same time point (**P<0.01; ***P<0.001).

It was noticed that the transcripts of two genes, namely Cit.17340.1.S1_at (*PtrPOD*) and Cit.17713.1.S1_s_at (*PtrADC*), were higher in the transgenic line #10 than in the WT, which raised the question of whether they were also induced in both transgenic lines and under dehydration conditions. The mRNA levels of *PtrPOD* and *PtrADC* were therefore examined in the two transgenic lines and the WT before and after dehydration. Transcript levels of the two genes in the transgenic lines were higher than in the WT under normal growth conditions. Dehydration for 90min increased their expression levels in both the WT and the transgenic lines; however, the degree of induction in the transgenic lines was more substantial than in the WT (Fig. 6a, b).

**Fig. 7.**
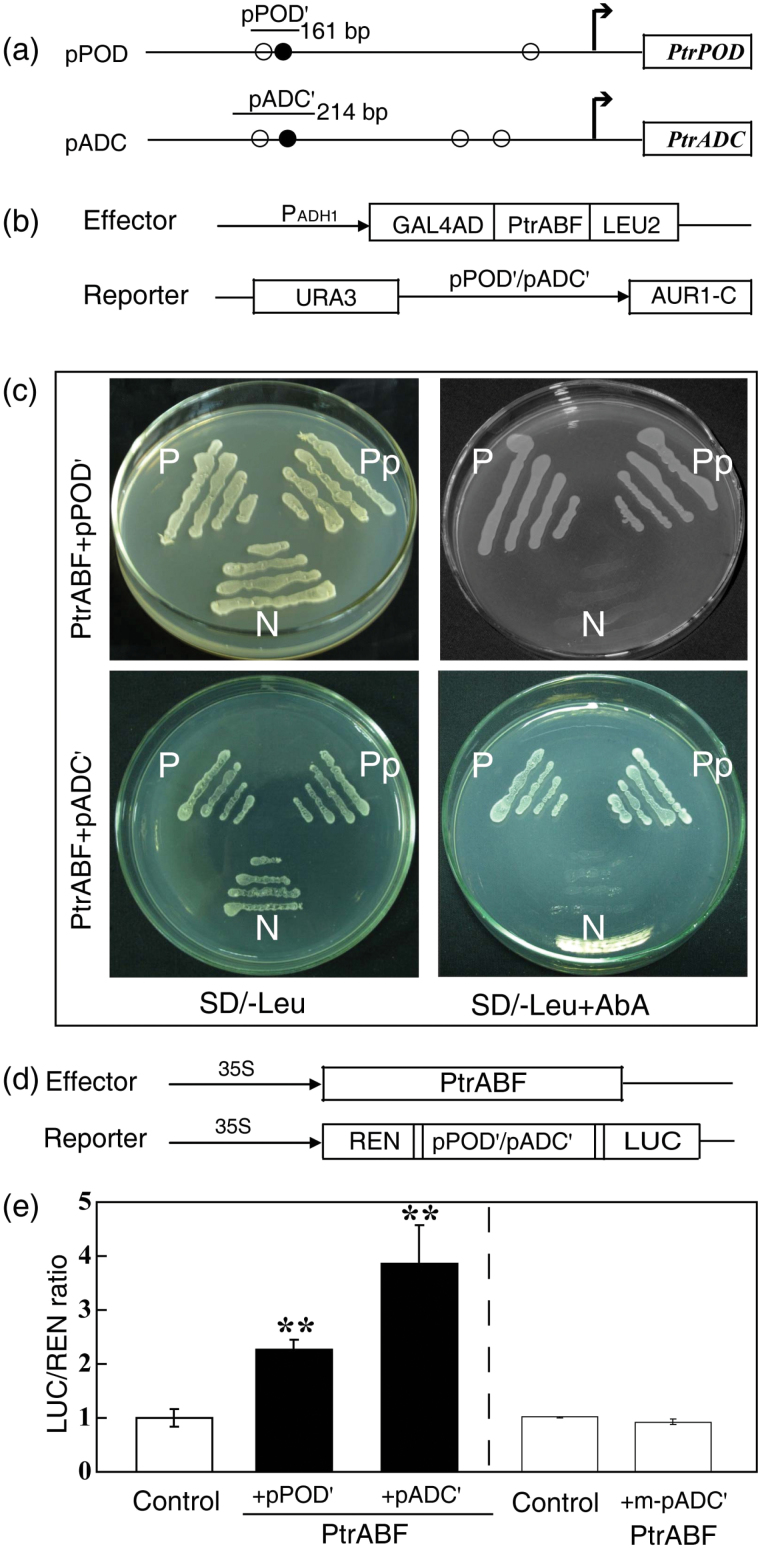
Interaction between PtrABF and the promoters of *PtrPOD* and *PtrADC*. (a) Schematic diagrams of the promoter of *POD* (pPOD) and *ADC* (pADC). The filled and open circles are ABREs and coupling elements (CEs), respectively. The short lines with pPOD’ or pADC’ show the partial promoter fragments used for the analysis. (b) The effector and reporter vectors used for yeast one-hybrid assay. (c) Growth of yeast cells of the positive control (P; p53-AbAi+pGAD-p53), negative control (N; pPOD’-AbAi+pGADT7 in the upper panels, pADC’-AbAi+pGADT7 in the bottom panels), and the effector–reporter co-transformant (Pp) on SD/-Leu medium without (left) or with (right) addition of 300mM AbA. (d) Schematic diagrams of the effector and reporter constructs used for transient expression assay. *PtrABF* driven by the CaMV *35S* promoter was used as the effector. In the reporter construct, the *POD* or *ADC* promoter fragments (pPOD’, pADC’) were fused to the upstream region of the *LUC* gene. The *REN* gene under the control of the CaMV *35S* promoter was used as a control for activity normalization. (e) Transient expression assay of transcriptional activation of *POD* and *ADC* promoters by PtrABF based on the relative LUC activities in tobacco protoplasts co-transformed with the effector and the reporter. LUC/REN ratio of the control (in the absence of the effector, –PtrABF) was taken as 1, while co-transformation of PtrABF and a promoter fragment (m-pADC’) without an ABRE element was used to examine specificity. Asterisks indicate that the values are significantly different from each other (**P<0.01).

*In silico* analysis showed that the *PtrPOD* promoter has one ABRE and two CEs, while the *PtrADC* promoter has one ABRE and three CEs (Fig. 7a). Given that the transcript levels of both *PtrPOD* and *PtrADC* were higher in the transgenic lines, it was reasoned that they may be downstream targets of *PtrABF*. To verify this, the interaction of PtrABF with the *PtrPOD* and *PtrADC* promoters was investigated using a Y1H analysis and a transient expression assay. For the Y1H analysis, the *PtrABF* coding sequence was fused with GAL4 to generate the effector construct, while the promoter fragments of *PtrPOD* (pPOD’) and *PtrADC* (pADC’) containing one ABRE and one CE were fused with pAbAi to create the reporter vector (Fig. 7b). The effector and each of the two reporter vectors were co-transformed into the yeast strain Y1HGold, which was spread on SD/-Leu medium or SD/-Leu supplemented with the antibiotic aureobasidin A (AbA). The co-transformant, positive control, and negative control grew well on the SD/-Leu medium, but only the co-transformant and positive control survived on the medium supplemented with AbA (Fig. 7c), suggesting that PtrABF interacted with the promoters of both *PtrPOD* and *PtrADC*.

The transient dual expression assay was also performed to verify the Y1H results. A reporter vector was generated by fusing pPOD’ or pADC’ to the N-terminus of the *Luciferase* gene (LUC), and *PtrABF* under the control of the CaMV *35S* promoter was used as the effector (Fig. 7d). Each of the reporter and the effector vectors was transferred into tobacco protoplasts, and the expression ratio of LUC/REN (Renilla luciferase) was measured and calculated. Compared with the control, co-transformation with the effector (PtrABF) significantly elevated reporter activity (Fig. 7e), indicating that PtrABF can activate these two promoters. When PtrABF was co-transformed with a promoter sequence (m-pADC’) that did not contain an ABRE, the reporter activity was not changed relative to the control, indicating the specificity of promoter activation by PtrABF.

### Transgenic plants have higher antioxidant enzyme activities and polyamine levels, but a lower abundance of ROS

**Fig. 8.**
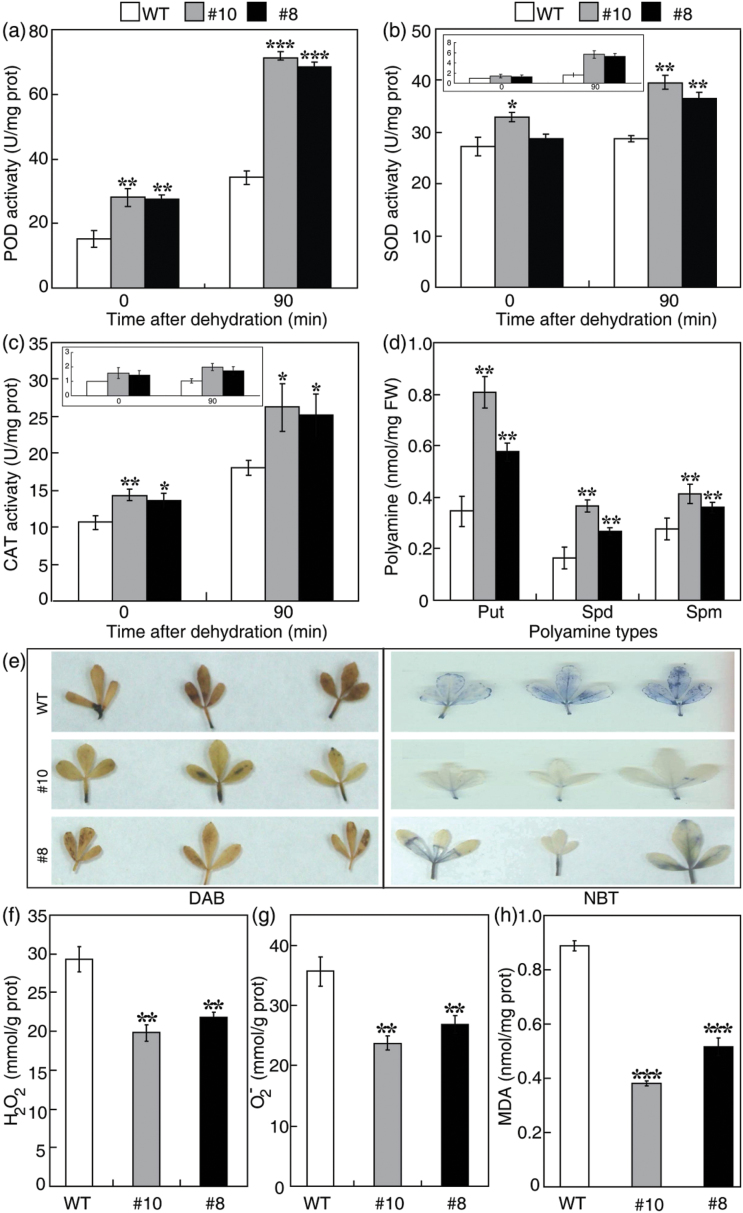
The transgenic lines exhibited higher antioxidant enzyme activity and polyamine levels, but lower levels of ROS and MDA. (a–c) Activities of POD (a), SOD (b), and CAT (c) in the wild type (WT) and transgenic lines (#8, #10) before and after dehydration treatment. Error bars indicate the SD (*n*=3). The insets in b and c indicate transcript levels of *SOD* and *CAT* genes before and after dehydration treatment. (d) Free polyamine contents in the WT and transgenic lines under normal growth conditions. Error bars indicate the SD (*n*=3). (e) Accumulation of H_2_O_2_ (left panels) and O_2_^–^ (right panels) in the WT and transgenic lines after dehydration treatment, as revealed by histochemical staining with DAB and NBT, respectively. (f–h) Quantitative measurement of H_2_O_2_ (f), O_2_^–^ (g), and MDA (h) in the WT and transgenic lines after dehydration. Asterisks indicate significant differences between the transgenic lines and the WT at the same time point (*P<0.05, **P<0.01, ***P<0.001).

Using the transcriptomic data as a guide, the physiological mechanisms underlying the enhanced stress tolerance of the transgenic lines were investigated. Specifically, the activities of antioxidant enzymes, including POD, catalase (CAT), and superoxide dismutase (SOD) were examined, and the levels of polyamines were also measured. POD activity in the transgenic lines was approximately twice that of the WT before and after dehydration (Fig. 8a). The activities of SOD and CAT in the transgenic lines were slightly higher than in the WT without stress treatment, consistent with the expression of the *SOD* and *CAT* genes. The dehydration treatment caused an increase in these enzyme activities in both transgenic and WT plants, but the former had significantly higher activity levels (Fig. 8b, c). The levels of three free polyamines, namely putrescine, spermidine, and spermine, were significantly higher in the transgenic lines than in the WT before dehydration (Fig. 8d). Exposure to dehydration conditions for 90min caused minor changes in the levels of free polyamines; however, the transgenic lines still contained higher levels than WT plants.

Since the three antioxidant enzymes (POD, CAT, and SOD) are known to play key roles in ROS scavenging, the accumulation of H_2_O_2_ and O_2_^–^ in the dehydrated leaves was assessed by histochemical staining using DAB (3,3′-diaminobenzidine) and NBT (nitroblue tetrazolium), respectively. The WT leaves displayed deeper staining than leaves from the transgenic lines (Fig. 8e), indicating that the latter had lower levels of ROS under dehydration. The histochemical analysis was further supported by quantitative measurements of H_2_O_2_ and O_2_^–^ (Fig. 8f, g). The levels of MDA, a product of lipid peroxidation, in the dehydrated leaves were also measured. Consistent with the observed lower ROS accumulation, MDA levels in the leaves of the two transgenic lines were substantially lower than in the WT after the dehydration treatment (Fig. 8h). Taken together, the results indicate that the transgenic lines have a greater capacity for ROS scavenging and accumulate lower levels of ROS and MDA. However, when the H_2_O_2_ level in the guard cells was monitored using H2DCF-DA, a fluorescent dye, no substantial difference was detected between the transgenic lines and the WT (Supplementary Fig. S4 at *JXB* online).

### d-Arginine and guazatine affect ROS accumulation as well as the expression and activity of antioxidant enzymes

**Fig. 9.**
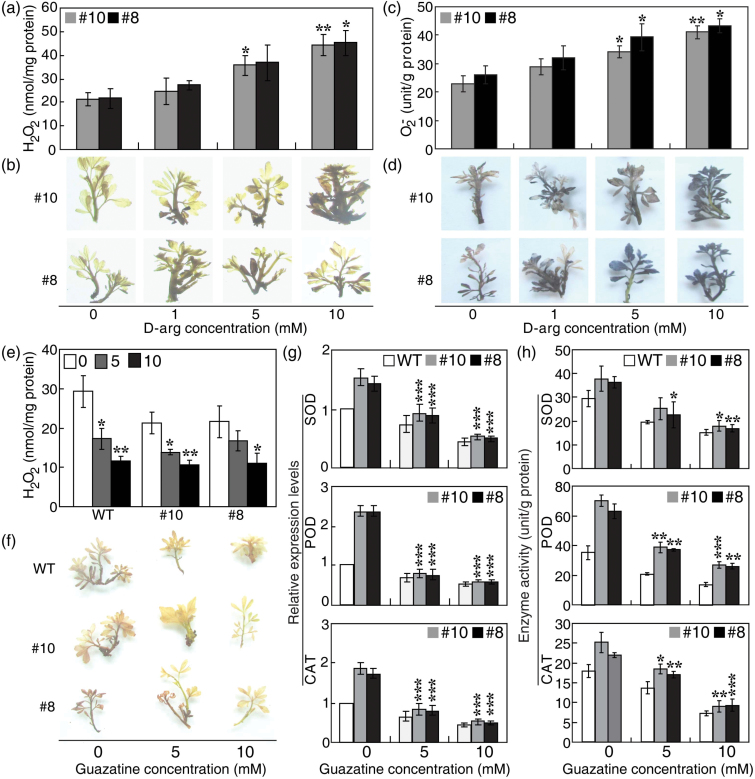
d-Arginine (d-arg) and guazatine treatments altered ROS accumulation, activity, and transcripts of antioxidant enzymes. (a–d) Treatment with d-arg increased ROS accumulation in the transgenic lines (#8, #10) after dehydration stress. The transgenic lines were pre-treated with water (0mM) or 1, 5, and 10mM d-arg for 3 d prior to 90min of dehydration. Accumulation of H_2_O_2_ (a, b) and O_2_^–^ (c, d) was quantitatively measured (a, c) or histochemically detected using DAB (b, for H_2_O_2_) and NBT (d, for O_2_^–^). (e, f) Guazatine treatment reduced the accumulation of H_2_O_2_ in the wild type (WT) and transgenic lines after dehydration. The transgenic lines and the WT were pre-treated with water (0mM) or 5mM and 10mM guazatine for 3 d before they were exposed to 90min of dehydration. Accumulation of H_2_O_2_ was quantitatively measured (e) or histochemically detected using DAB staining (f). (g, h) Expression levels (g) and activities (h) of the antioxidant enzymes SOD, POD, and CAT in transgenic lines and the WT pre-treated with water (0mM) or 5mM and 10mM guazatine. Asterisks indicate significant differences between inhibitor treatment and water treatment of the same line (*P<0.05, **P<0.01, ***P<0.001).

It was next investigated whether the elevated polyamine levels might play a role in modulating ROS accumulation in the *PtrABF*-overexpressing lines. The transgenic lines were pre-treated with different concentrations of d-arginine, an inhibitor of ADC, prior to the dehydration treatment. Compared with control samples treated with water, levels of both H_2_O_2_ and O_2_^–^ were increased in the d-arginine-treated samples, in a dosage-dependent manner; an observation that was supported by both quantitative measurements and histochemical staining (Fig. 9a–d).

Polyamine catabolism mediated by polyamine oxidase (PAO; EC 1.5.3.3) results in the production of H_2_O_2_ (Moschou *et al.*, 2008*a*, *b*; Miller *et al.*, 2010). It was thus asked whether PAO-mediated generation of H_2_O_2_ contributed to the ROS accumulation. WT and transgenic lines were treated with guazatine, a PAO inhibitor, prior to dehydration treatment. Both quantitative measurements (Fig. 9e) and histochemical staining (Fig. 9f) demonstrated that H_2_O_2_ levels in the WT and transgenic lines pre-treated with guazatine were lower than in the water-treated controls. It was observed that higher concentrations of guazatine led to a more substantial reduction in H_2_O_2_ levels. However, the transgenic lines still accumulated less H_2_O_2_ relative to the WT. In addition, it was observed that the transcript levels and activities of the antioxidant enzymes SOD, POD, and CAT were reduced in the guazatine-treated samples (Fig. 9g, h), consistent with the changes in H_2_O_2_ levels.

## Discussion

Over the last decade, significant progress has been made with respect to our understanding of the ABA-mediated signalling network. For example, ABFs are now regarded as key regulators associated with adaptive stress responses, reflecting their role in the transduction of the upstream stress signals to the downstream target genes. Although earlier studies demonstrated that different ABFs may have overlapping functions, each also appears to have a specific role in ABA-mediated signalling in response to abiotic stresses (Yoshida *et al.*, 2010), so specific ABFs may regulate different webs of target genes. Even though the functions of a few ABFs have been extensively studied, the target genes of ABF4 are still poorly characterized so far. Therefore, deciphering target genes of ABFs is of paramount significance to better understand their mode of action in stress response.

In the present study, it is demonstrated that overexpression of *PtrABF* confers enhanced dehydration tolerance, as shown by reduced water loss and less serious leaf wilting in transgenic plants overexpressing the gene. Plants have developed a variety of mechanisms to achieve dehydration tolerance, some of which involve alterations of stomatal properties, including aperture size and stomatal density. Since transpiration through stomata accounts for the majority of water loss, the regulation of these properties is critical for limiting water loss in response to drought stress (Nilson and Assmann, 2007; Xie *et al.*, 2012). In this study, it was noticed that the stomatal apertures of *PtrABF*-overexpressing transgenic lines were slightly smaller than those of the WT with or without an exposure to dehydration stress. This finding is congruent with previous studies (Kang *et al.*, 2002; Cominelli *et al.*, 2010) and suggests that enhanced dehydration tolerance of the *ABF*-overexpressing lines may be partly attributed to reduced stomatal apertures. However, this contribution might be negligible as the difference in stomatal apertures between the WT and transgenic lines was minute when compared with the prominent difference in stomatal density, which was remarkably reduced in the transgenic lines compared with the WT. These data suggest that reduction of the stomatal density might be the major reason why the transgenic lines displayed improved water retention under dehydration stress. In addition, it is tempting to assume that stomatal development rather than physiology was primarily influenced by *PtrABF* overexpression. *PtrABF* thus joins the list of genes that have been reported to alter stomatal density, such as *MtCAS31* (Xie *et al.*, 2012), *WRKY20* (Luo *et al.*, 2013), and *AtTRE1* (Van Houtte *et al.*, 2013). It was also noticed that the transgenic lines displayed substantially lower stomatal indices compared with the WT, indicating that fewer pavement cells of the transgenic leaves entered the stomatal development pathway and continued to develop stomata. Stomatal development is regulated by environmental cues, such as light and carbon dioxide levels (Casson and Hetherington, 2010), and significant advances have also been made in our understanding of the transcriptional regulation of stomatal development. Stomata are generated through a series of differentiation events mediated by three closely related bHLH TFs, SPCH, MUTE, and FAMA. These three proteins act as positive regulators controlling entry into the stomatal cell lineage, the transition from a meristemoid to a guard mother cell, and the final differentiation of guard cells, respectively (MacAlister *et al.*, 2007; Pillitteri *et al.*, 2007; Casson and Hetherington, 2010). A recent study demonstrated that ICE1 physically interacts with the three bHLH proteins and is necessary for stomatal differentiation (Kanaoka *et al.*, 2008). In the core regulatory unit, the formation of heterodimers between ICE1 and each of the three bHLH TFs plays a crucial role in specifying the cell state transitional steps of stomatal development. It was shown in this study that PtrABF can physically interact with PtrICE1. However, the interaction between PtrABF and PtrICE1 in the cytoplasm of leaf epidermal cells was unexpected as both of them are TFs. It is assumed that this interaction may possibly prevent movement of PtrICE1 into the nucleus and thus inhibits the generation of biologically functional dimers between PtrICE1 and the three bHLH proteins, leading to a repression of the stomatal lineage. Although more work is required to clarify this, the assumption is in part supported by the decrease in stomatal indices of the transgenic lines. However, whether and how this interaction caused reduction of transcript levels of the bHLH genes in the transgenic lines remained elusive. According to this model, sequential steps of stomatal differentiation in the transgenic plants were impaired, resulting in the observed decrease in stomatal index. This finding provides new insights into the function of an *ABF* gene in stomatal development, although the underlying mechanisms remain to be determined.

The genome-wide microarray analysis revealed a minor change in the transcriptome of the transgenic line overexpressing *PtrABF*, with 42 and 28 genes being induced and repressed, respectively. It is worth mentioning that the microarrays did not have probes for the three bHLH genes, namely *PtrSPCH*, *PtrMUTE*, and *PtrFAMA*. As PtrABF has been shown to act as a transactivator, the focus of this study was primarily on the genes that were induced in the transgenic line. A small number of the induced genes encode regulatory proteins, including several TFs, such as NAC, WRKY, and bZIP, which are known to function as regulators of abiotic stress responses (Liu *et al.*, 2014). Moreover, the majority of the DEGs were categorized by GO analysis as functional genes that play roles in defence against abiotic stresses. The induction of these stress-associated functional or regulatory genes may be largely responsible for the improved dehydration tolerance of the transgenic plants.

The microarray analysis also suggested that a number of genes might be directly regulated by PtrABF. *In silico* analysis revealed that the promoters of 28 induced DEGs contained various combinations of ABREs and CEs. Of these, 23 gene promoters had the Type I and Type II combinations, making them more probable direct targets of PtrABF. This reasoning is dependent upon the notion that a minimal sequence composed of at least two copies of ABREs and CEs is necessary and sufficient to guarantee the transcriptional regulation of an ABF protein and confer ABA response (Choi *et al.*, 2000). The present results concerning the interaction between PtrABF and the promoter fragments containing the minimal sequence provide experimental evidence supporting the regulation of these genes by PtrABF. This regulation may in turn cause the observed increase in transcript levels of the target genes in the transgenic line. Interestingly, it was also found that five annotated DEGs induced in the transgenic line contained neither an ABRE nor a CE, while another five (Type III) had only one ABRE or CE. Based on the prerequisite of a functional minimal sequence, they may be excluded from the group of direct PtrABF targets. One possibility for the observed induction of these genes in the transgenic line is that they are regulated by TFs among the 23 genes with Type I or Type II promoters. Interestingly, the potential targets include a gene in the LEA family that has previously been identified as a possible target of ABF3 and ABF2 (Fujita *et al.*, 2005; Oh *et al.*, 2005), indicating that LEA genes may be regulated by multiple ABFs. The LEAs are hydrophilic proteins that are widely assumed to play critical roles in cellular dehydration tolerance by protein stabilization, enzyme protection, and membrane association and stabilization (Fujita *et al.*, 2005; Olvera-Carrillo *et al.*, 2011). Activation of the LEA gene in this study suggests that maintenance of favourable cell turgidity may also contribute to the enhanced dehydration/drought tolerance in the *ABF*-overexpressing plants.

The presence of a common set of target genes also supports the previous notion that ABFs have overlapping functions in response to water stress (Yoshida *et al.*, 2010). However, the target genes identified in the present study are largely different from those in earlier studies, and some of the genes identified here have not been previously suggested as potential targets. Two examples are *PtrPOD* and *PtrADC*, which have however previously been shown to be involved in stress tolerance responses (Wang *et al.*, 2011; Huang *et al.*, 2013; Shi and Chan, 2014), and identification of these genes as ABF targets expands the regulon of ABFs. This in turn provides valuable clues to elucidate the mechanisms underlying the enhanced stress tolerance in the *PtrABF*-overexpressing lines. It is also noted that the identification of *PtrABF* as a positive regulator of *PtrADC* offers a line of evidence to explain the accumulation of putrescine in many plant species exposed to drought treatments (Liu *et al.*, 2007; Shi and Chan, 2014, and references therein). It is proposed that under drought stress ABA accumulates and triggers the well-known signalling pathway from ABA perception to SnRK activation (Nakashima and Yamaguchi-Shinozaki, 2013; Yoshida *et al.*, 2015). The activated SnRKs then phosphorylate ABF4, which in turn induces *ADC*, resulting in the production of active enzymes involved in putrescine synthesis.

Exposure to abiotic stresses leads to the elevated generation of ROS, causing oxidative damage to cellular components, which is considered a primary factor in cellular injury in plants (Kasukabe *et al.*, 2004). ROS accumulation is dependent on the balance between scavenging and production. Plants have developed a conserved defence system to maintain ROS homeostasis under unfavourable environmental conditions. For example, antioxidant enzymes play an essential protective role in ROS scavenging (Miller *et al.*, 2010). In this work, transgenic plants had higher activities of three antioxidant enzymes (CAT, POD, and SOD), suggesting that they possess a more efficient enzymatic antioxidant system compared with the WT. As a result, ROS generated in the transgenic plants would probably be scavenged more easily. This conclusion is supported by the substantially reduced levels of H_2_O_2_ and O_2_^–^ in the transgenic lines under dehydration stress, accompanied by an alleviation of lipid peroxidation (lower MDA content). Taken together, these results indicate that the transgenic plants have a more potent ROS sequestration machinery, allowing them to better withstand ROS-associated oxidative stress. It is proposed that the activation of ROS-scavenging enzymes constitutes an important physiological mechanism underlying the promotion of dehydration tolerance by *PtrABF*.

Polyamines are low molecular weight aliphatic amines ubiquitously present in living organisms. Being polycationic, polyamines can bind to anionic molecules, such as nucleic acids, proteins, and molecules with phosphate head-groups. This attribute, together with their potential as compatible solutes, underpins their role in combating abiotic stresses through stabilization of cellular membranes and key macromolecules, or mitigation of osmotic stress (Kusano *et al.*, 2008; Minocha *et al.*, 2014; Tiburcio *et al.*, 2014). In this study, the transgenic lines were observed to contain higher levels of polyamines, which may help ameliorate any damage caused by dehydration stress. The present results agree with earlier studies showing that high levels of polyamines correlate with enhanced stress tolerance (Liu *et al.*, 2007; Alcázar *et al.*, 2010; Tiburcio *et al.*, 2014). In contrast, a growing body of evidence demonstrated that polyamines of higher molecular weight, including spermidine and spermine, are subjected to PAO-mediated catabolism when their levels increase beyond a threshold, leading to production of various metabolites and H_2_O_2_ (Moschou *et al.*, 2008*a*, *b*; Tiburcio *et al.*, 2014). The *PtrABF*-overexpressing plants might be expected to reach the threshold value earlier than the WT because they contained higher levels of endogenous polyamines, leading to earlier initiation of PA catabolism in the transgenic lines. At the early stage of stress, PAO-mediated polyamine catabolism possibly resulted in a limited amount of H_2_O_2_, which has been suggested to act as a potent signalling molecule at low levels, to orchestrate stress responses by activating downstream defence components (Huang *et al.*, 2005). Since H_2_O_2_ has been reported to function as an intermediate in ABA signalling (Zhang *et al.*, 2001; An *et al.*, 2008), polyamines might play an indirect role in ABA-dependent signal transduction associated with stress responses. Accordingly, extensive changes in the transcript levels of ABA-dependent genes have been observed in plants with an altered pool of endogenous polyamines. For instance, transgenic *A. thaliana* plants overexpressing a spermidine synthase gene from *Cucurvita ficifolia* display increased expression of a range of ABA-dependent transcription factors and functional proteins (Kasukabe *et al.*, 2004), and putrescine has been shown to interact with ABA-dependent signalling pathways (Cuevas *et al.*, 2008). Therefore, in the current study, the higher polyamine levels in the transgenic plants might modulate the ABA signalling network, which involves a large spectrum of stress-related genes, including antioxidant enzymes (Xian *et al.*, 2014). Polyamines can thus potentially influence enzymatic antioxidant systems through a H_2_O_2_-mediated signalling cascade and in turn facilitate ROS elimination. This H_2_O_2_-driven signalling may contribute greatly to the induction of SOD and CAT genes, which, even though they were not identified here as PtrABF targets, were prominently induced in the transgenic plants. This hypothesis is in part supported by a decrease in antioxidant enzyme activities and higher ROS production in the tissues treated with guazatine. On the other hand, PtrABF-mediated signalling may intersect with H_2_O_2_ signalling to exert a synergistic effect on the antioxidant enzymes. The involvement of polyamines in ROS elimination was further suggested by an experiment where the transgenic plants were treated with d-arginine, thus corroborating several earlier reports on the activation of antioxidant enzymes and the promotion of ROS detoxification by elevating endogenous polyamine contents (Wen *et al.*, 2010; Jang *et al.*, 2012). Collectively, these results indicate that overexpression of *PtrABF* led to elevated levels of endogenous polyamines, which act either as direct cellular guard metabolites or as substrates for generating H_2_O_2_ signalling. However, it is worth mentioning that the H_2_O_2_ levels in the guard cells were nearly equivalent between WT and transgenic lines, which suggests that the polyamine-derived H_2_O_2_ may play negligible role in stomatal movement or that the transgene effects may be limited to certain tissues.

Increasing the duration and intensity of the dehydration stress resulted in the production of a large amount of H_2_O_2_ and other types of ROS in both the transgenic lines and the WT. These in turn served as inducers of oxidative stresses and, consequently, cellular damage, rather than acting as signalling molecules as they did at lower levels. In this study, treatment with an inhibitor of PAO led to a substantial reduction in H_2_O_2_ levels at the end of the dehydration treatment, indicating that PAO-mediated H_2_O_2_ contributed greatly to the overall ROS homeostasis under dehydration conditions. Several earlier studies demonstrated that PAO activity remains fairly constant irrespective of alterations in the polyamine pool if endogenous PAO is not artificially modified (Moschou *et al.*, 2008*a*; Alcázar *et al.*, 2011). It is speculated that large amounts of ROS may be generated by PAO catabolism in the transgenic lines and the WT after exposure to severe dehydration stress. However, as enzymatic antioxidant systems were more highly induced in the transgenic lines than in the WT, ROS produced in the former were more efficiently removed. In contrast, ROS in the WT were not properly quenched, leading to stronger oxidative stress and cell death. These results reinforce the view that elevation of polyamine anabolism, in the presence of constant polyamine catabolism, increases the polyamine anabolism to catabolism ratio and reduces the detrimental effects of ROS, leading to enhanced stress tolerance (Moschou *et al.*, 2008*a*; Tiburcio *et al.*, 2014).

**Fig. 10.**
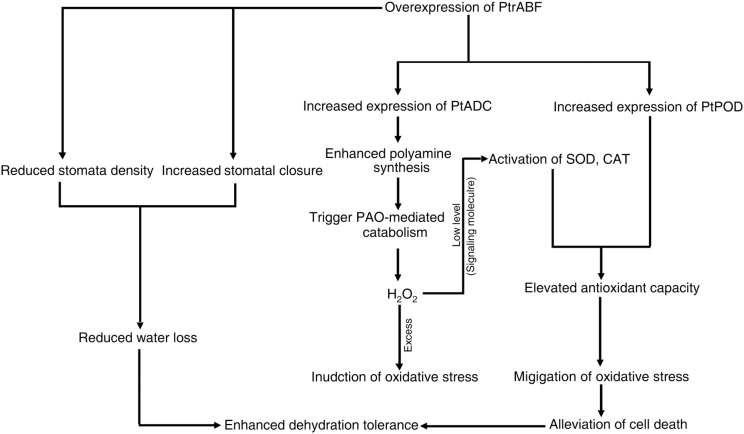
A proposed mode of action of *PtrABF* based on the current study. In this model, *PtrABF* mediates dehydration tolerance via two major mechanisms: (1) mitigation of water loss by stimulating stomatal closure and decreasing stomatal density through interacting with PtrICE1; and (2) transcriptional regulation of target genes encoding enzymes associated with antioxidant (*POD*) or polyamine synthesis (*ADC*), by interacting with ABRE *cis*-acting elements. Higher levels of polyamines trigger catabolism mediated by PAO. This generates low levels of H_2_O_2_ at the initial stage of stress, which serves as a signalling molecule that activates stress-associated genes, including those encoding antioxidant enzymes. As a result, antioxidant capacity is enhanced, which in turn expedites detoxification of excess ROS and leads to a reduction in the oxidative stress and the alleviation of cell death.

In summary, overexpression of *PtrABF* led to enhanced dehydration tolerance. Based on the data reported here and in earlier studies (Cuevas *et al.*, 2008; Moschou *et al.*, 2008*a*, *b*; Alcázar *et al.*, 2011; Tiburcio *et al.*, 2014), a possible mode of action of *PtrABF* is suggested (Fig. 10). First, *PtrABF* stimulates stomatal closure and decreases stomatal density, leading to an alleviation of water loss. Secondly, *PtrABF* positively regulates the expression of an array of target genes, some of which encode key enzymes involved in antioxidant systems and polyamine synthesis. The products of these genes may function to eliminate ROS at lethal dosages, either directly (antioxidant enzymes) or indirectly (polyamines, serving as a substrate producing a signalling molecule), and act as protective metabolites (polyamines). The results of the current study further the understanding of the physiological and molecular mechanisms underlying ABF-mediated stress tolerance.

## Supplementary data

Supplementary data are available at *JXB* online.

Figure S1. Generation and molecular identification of trifoliate orange transgenic plants overexpressing *PtrABF*.

Figure S2. Comparison of dehydration tolerance between wild-type and transgenic plants containing the *NPTII* gene.

Figure S3. Subcellular localization of PtrABF and PtrICE1.

Figure S4. Analysis of H_2_O_2_ in guard cells.

Table S1. Primer sequences used for this study.

Table S2. Comparison of stomatal development genes between *Poncirus trifoliata* and *Arabidopsis thaliana*.

Table S3. A list of genes repressed in transgenic line #10 overexpressing *PtrABF* in comparison with the wild type, as revealed by microarray analysis.

Supplementary methods. Detailed methods used for this study.

Supplementary Data

## References

[CIT0001] Alcázar R Bitrián M Bartels D Koncz C Altabella T Tiburcio AF 2011 Polyamine metabolic canalization in response to drought stress in Arabidopsis and the resurrection plant *Craterostigma plantagineum* . Plant Signaling and Behavior 6, 243–250. 21330782 10.4161/psb.6.2.14317 PMC3121985

[CIT0002] Alcázar R Altabella T Marco F Bortolotti C Reymond M Koncz C Carrasco P Tiburcio AF 2010 Polyamines: molecules with regulatory functions in plant abiotic stress tolerance. Planta 231, 1237–1249. 20221631 10.1007/s00425-010-1130-0

[CIT0003] An Z Jing W Liu Y Zhang W 2008 Hydrogen peroxide generated by copper amine oxidase is involved in abscisic acid-induced stomatal closure in *Vicia faba* . Journal of Experimental Botany 59, 815–825. 18272918 10.1093/jxb/erm370

[CIT0004] Casson SA Hetherington AM 2010 Environmental regulation of stomata development. Current Opinion in Plant Biology 13, 90–95. 19781980 10.1016/j.pbi.2009.08.005

[CIT0005] Choi H Hong JH Ha JO Kang JY Kim SY 2000 ABFs, a family of ABA-responsive element binding factors. Journal of Biological Chemistry 275, 1723–1730. 10636868 10.1074/jbc.275.3.1723

[CIT0006] Cominelli E Galbiati M Tonelli C 2010 Transcription factors controlling stomatal movements and drought tolerance. Transcription 1, 41–45. 21327157 10.4161/trns.1.1.12064 PMC3035188

[CIT0007] Cuevas JC López-Cobollo R Alcázar R Zarza X Koncz C Altabella T Salinas J Tiburcio AF Ferrando A 2008 Putrescine is involved in Arabidopsis freezing tolerance and cold acclimation by regulating abscisic acid levels in response to low temperature. Plant Physiology 148, 1094–1105. 18701673 10.1104/pp.108.122945 PMC2556839

[CIT0008] Danquah A de Zelicout A Colcombet J Hirt H 2014 The role of ABA and MAPK signaling pathways in plant abiotic stress responses. Biotechnology Advance 32, 40–52. 10.1016/j.biotechadv.2013.09.006 24091291

[CIT0009] Fu XZ Khan EU Hu SS Fan QJ Liu JH 2011 Overexpression of the betaine aldehyde dehydrogenase gene from *Atriplex hortensis* enhances salt tolerance in the transgenic trifoliate orange (*Poncirus trifoliata* (L.) Raf.). Environmental and Experimental Botany 74, 106–113.

[CIT0010] Fujita Y Fujita M Satoh R Maruyama K Parvez MM Seki M Hiratsu K Ohme-Takagi M Shinozaki K Yamaguchi-Shinozaki K 2005 AREB1 is a transcription activator of novel ABRE-dependent ABA signaling that enhances drought stress tolerance in Arabidopsis. The Plant Cell 17, 3470–3488. 16284313 10.1105/tpc.105.035659 PMC1315382

[CIT0011] Fujita Y Yoshida T Yamaguchi-Shinozaki K 2013 Pivotal role of the AREB/ABF–SnRK2 pathway in ABRE-mediated transcription in response to osmotic stress in plants. Physiologia Plantarum 147, 15–27. 22519646 10.1111/j.1399-3054.2012.01635.x

[CIT0012] Hossain MA Lee Y Cho JI Ahn CH Lee SK Jeon JS Kang H Lee CH An G Park PB 2010 The bZIP transcription factor *OsABF1* is an ABA responsive element binding factor that enhances abiotic stress signaling in rice. Plant Molecular Biology 72, 557–566. 20039193 10.1007/s11103-009-9592-9

[CIT0013] Hsieh TH Li CW Su RC Cheng CP Sanjaya Tsai YC Chan MT 2010 A tomato bZIP transcription factor, *SlAREB*, is involved in water deficit and salt stress response. Planta 231, 1459–1473. 20358223 10.1007/s00425-010-1147-4

[CIT0014] Huang SH Yu CW Lin CH 2005 Hydrogen peroxide functions as a stress signal in plants. Botanical Bulletin of Academia Sinica 46, 1–10.

[CIT0015] Huang XS Liu JH Chen XJ 2010 Overexpression of *PtrABF* gene, a bZIP transcription factor isolated from *Poncirus trifoliata*, enhances dehydration and drought tolerance in tobacco via scavenging ROS and modulating expression of stress-responsive genes. BMC Plant Biology 10, 230. 20973995 10.1186/1471-2229-10-230 PMC3017851

[CIT0016] Huang XS Wang W Zhang Q Liu JH 2013 A basic helix–loop–helix transcription factor *PtrbHLH*, of *Poncirus trifoliata* confers cold tolerance and modulates peroxidase-mediated scavenging of hydrogen peroxide. Plant Physiology 162, 1178–1194. 23624854 10.1104/pp.112.210740 PMC3668048

[CIT0017] Jang SJ Wi SJ Choi YJ An G Park KY 2012 Increased polyamine biosynthesis enhances stress tolerance by preventing the accumulation of reactive oxygen species: T-DNA mutational analysis of *Oryza sativa* lysine decarboxylase-like protein1. Molecules and Cells 34, 251–262. 22965749 10.1007/s10059-012-0067-5 PMC3887846

[CIT0018] Kanaoka MM Pillitteri LJ Fujii H Yoshida Y Bogenschutz NL Takabayashi J Zhu JK Torii KU 2008 *SCREAM/ICE1* and *SCREAM2* specify three cell-state transitional steps leading to *Arabidopsis* stomatal differentiation. The Plant Cell 20, 1775–1785. 18641265 10.1105/tpc.108.060848 PMC2518248

[CIT0019] Kasukabe Y He L Nada K Misawa S Ihara I Tachibana S 2004 Overexpression of spermidine synthase enhances tolerance to multiple environmental stresses and up-regulates the expression of various stress-regulated genes in transgenic *Arabidopsis thaliana* . Plant and Cell Physiology 45, 712–722. 15215506 10.1093/pcp/pch083

[CIT0020] Kang JY Choi HI Im MY Kim SY 2002 Arabidopsis basic leucine zipper proteins that mediate stress-responsive abscisic acid signaling. The Plant Cell 14, 343–357. 11884679 10.1105/tpc.010362 PMC152917

[CIT0021] Kim JB Kang JY Kim SY 2004 *a* Over-expression of a transcription factor regulating ABA-responsive gene expression confers multiple stress tolerance. Plant Biotechnology Journal 2, 459–466. 17168892 10.1111/j.1467-7652.2004.00090.x

[CIT0022] Kim S Kang JY Cho DI Park JH Kim SY 2004 *b* ABF2, an ABRE-binding bZIP factor, is an essential component of glucose signaling and its overexpression affects multiple stress tolerance. The Plant Journal 40, 75–87. 15361142 10.1111/j.1365-313X.2004.02192.x

[CIT0023] Kim SY 2006 The role of ABF family bZIP class transcription factors in stress response. Physiologia Plantarum 126, 519–527.

[CIT0024] Kusano T Berberich T Tateda C Takahashi Y 2008 Polyamines: essential factors for growth and survival. Planta 228, 367–381. 18594857 10.1007/s00425-008-0772-7

[CIT0025] Liu JH Kitashiba H Wang J Ban Y Moriguchi T 2007 Polyamines and their ability to provide environmental stress tolerance to plants. Plant Biotechnology 24, 117–126.

[CIT0026] Liu JH Moriguchi T 2007 Changes in free polyamine titers and expression of polyamine biosynthetic genes during growth of peach *in vitro* callus. Plant Cell Reports 26, 125–131. 16912865 10.1007/s00299-006-0223-5

[CIT0027] Liu JH Peng T Dai WS 2014 Critical *cis*-acting elements and interacting transcription factors: key players associated with abiotic stress responses in plants. Plant Molecular Biology Reporter 32, 303–317.

[CIT0028] Livak KJ Schmittgen TD 2001 Analysis of relative gene expression data using real-time quantitative PCR and the 2^–∆∆ C(T)^ method. Methods 25, 402–408. 11846609 10.1006/meth.2001.1262

[CIT0029] Luo X Bai X Sun X 2013 Expression of wild soybean *WRKY20* in *Arabidopsis* enhances drought tolerance and regulates ABA signaling. Journal of Experimental Botany 64, 2155–2169. 23606412 10.1093/jxb/ert073

[CIT0030] Ma Y Szostkiewicz I Korte A Moes D Yang Y Christmann A Grill A 2009 Regulators of PP2C phosphatase activity function as abscisic acid sensors. Science 324, 1064–1068. 19407143 10.1126/science.1172408

[CIT0031] MacAlister CA Ohashi-Ito K Bergmann DC 2007 Transcription factor control of asymmetric cell divisions that establish the stomatal lineage. Nature 445, 537–540. 17183265 10.1038/nature05491

[CIT0032] Mehrotra R Bhalothia P Bansal P Basantani MK Bharti V Mehrotra S 2014 Abscisic acid and abiotic stress tolerance—different tiers of regulation. Journal of Plant Physiology 171, 486–496. 24655384 10.1016/j.jplph.2013.12.007

[CIT0033] Miller G Suzuki N Ciftci-Yilmaz S Mittler R 2010 Reactive oxygen species homeostasis and signalling during drought and salinity stresses. Plant, Cell and Environment 33, 453–467. 10.1111/j.1365-3040.2009.02041.x 19712065

[CIT0034] Minocha R Majumdar R Minocha SC 2014 Polyamines and abiotic stress in plants: a complex relationship. Frontiers in Plant Science 5, 175. 24847338 10.3389/fpls.2014.00175 PMC4017135

[CIT0035] Moschou PN Paschalidis KA Delis ID Andriopoulou AH Lagiotis GD Yakoumakis DI Roubelakis-Angelakis KA 2008 *a* Spermidine exodus and oxidation in the apoplast induced by abiotic stress is responsible for H_2_O_2_ signatures that direct tolerance responses in tobacco. The Plant Cell 20, 1708–1724. 18577660 10.1105/tpc.108.059733 PMC2483379

[CIT0036] Moschou PN Sanmartin M Andriopoulou AH Rojo E Sanchez-Serrano JJ Roubelakis-Angelakis KA 2008 *b* Bridging the gap between plant and mammalian polyamine catabolism: a novel peroxisomal polyamine oxidase responsible for a full back-conversion pathway in Arabidopsis. Plant Physiology 147, 1845–1857. 18583528 10.1104/pp.108.123802 PMC2492618

[CIT0037] Muñiz García MN Giammaria V Grandellis C Téllez-Iñón MT Ulloa RM Capiati DA 2012 Characterization of StABF1, a stress-responsive bZIP transcription factor from *Solanum tuberosum* L. that is phosphorylated by StCDPK2 *in vitro* . Planta 235, 761–778. 22042328 10.1007/s00425-011-1540-7

[CIT0038] Nakashima K Yamaguchi-Shinozaki K 2013 ABA signaling in stress-response and seed development. Plant Cell Reports 32, 959–970. 23535869 10.1007/s00299-013-1418-1

[CIT0039] Nilson SE Assmann SM 2007 The control of transpiration. Insights from Arabidopsis. Plant Physiology 143, 19–27. 17210910 10.1104/pp.106.093161 PMC1761994

[CIT0040] Oh SJ Song SI Kim YS Jang HJ Kim SY Kim M Kim YK Nahm BH Kim JK 2005 Arabidopsis CBF3/DREB1A and ABF3 in transgenic rice increased tolerance to abiotic stress without stunting growth. Plant Physiology 138, 341–351. 15834008 10.1104/pp.104.059147 PMC1104188

[CIT0041] Olvera-Carrillo Y Reyes JL Covarrubias AA 2011 Late embryogenesis abundant proteins: versatile players in the plant adaptation to water limiting environments. Plant Signaling and Behavior 6, 586–589. 21447997 10.4161/psb.6.4.15042 PMC3142399

[CIT0042] Orellana S Yañez M Espinoza A Verdugo I González E Lara SR Casaretto JA 2010 The transcription factor SlAREB1 confers drought, salt stress tolerance and regulates biotic and abiotic stress-related genes in tomato. Plant, Cell and Environment 33, 2191–2208. 10.1111/j.1365-3040.2010.02220.x 20807374

[CIT0043] Park SY Fung P Nishimura N 2009 Abscisic acid inhibits type 2C protein phosphatases via the PYR/PYL family of START proteins. Science 324, 1068–1071. 19407142 10.1126/science.1173041 PMC2827199

[CIT0044] Pillitteri LJ Sloan DB Bogenschutz NL Torii KU 2007 Termination of asymmetric cell division and differentiation of stomata. Nature 445, 501–505. 17183267 10.1038/nature05467

[CIT0045] Pogány M von Rad U Grün S Dongó A Pintye A Simoneau P Bahnweg G Kiss L Barna B Durner J 2009 Dual roles of reactive oxygen species and NADPH oxidase RBOHD in an Arabidopsis–*Alternaria* pathosystem. Plant Physiology 151, 1459–1475. 19726575 10.1104/pp.109.141994 PMC2773049

[CIT0046] Shi H Chan Z 2014 Improvement of plant abiotic stress tolerance through modulation of the polyamine pathway. Journal of Integrative Plant Biology 56, 114–121. 24401132 10.1111/jipb.12128

[CIT0047] Shi J Fu XZ Peng T Huang XS Fan QJ Liu JH 2010 Spermine pretreatment confers dehydration tolerance of citrus *in vitro* plants via modulation of antioxidative capacity and stomatal response. Tree Physiology 30, 914–922. 20462936 10.1093/treephys/tpq030

[CIT0048] Shi Y Ding Y Yang S 2015 Cold signal transduction and its interplay with phytohormones during cold acclimation. Plant and Cell Physiology 56, 7–15. 25189343 10.1093/pcp/pcu115

[CIT0049] Shinozaki K Yamaguchi-Shinozaki K 2007 Gene networks involved in drought stress response and tolerance. Journal of Experimental Botany 58, 221–227. 17075077 10.1093/jxb/erl164

[CIT0050] Tiburcio AF Altabella T Bitrián M Alcázar R 2014 The roles of polyamines during the lifespan of plants: from development to stress. Planta 240, 1–18. 24659098 10.1007/s00425-014-2055-9

[CIT0051] Uno Y Furuhata T Abe H Yoshida R Shinozaki K Yamaguchi-Shinozaki K 2000 Arabidopsis basic leucine zipper transcription factors involved in an abscisic acid-dependent signal transduction pathway under drought and high-salinity conditions. Proceedings of the National Academy of Sciences, USA 97, 11632–11637. 10.1073/pnas.190309197 PMC17252 11005831

[CIT0052] Van Houtte H Vandesteene L López-Galvis L 2013 Overexpression of the trehalase gene *AtTRE1* leads to increased drought stress tolerance in arabidopsis and is involved in abscisic acid-induced stomatal closure. Plant Physiology 161, 1158–1171. 23341362 10.1104/pp.112.211391 PMC3585587

[CIT0053] Vanjildorj E Bae TW Riu KZ Kim SY Lee HY 2005 Overexpression of Arabidopsis ABF3 gene enhances tolerance to drought and cold in transgenic lettuce (*Lactuca sativa*). Plant Cell, Tissue and Organ Culture 83, 41–50.

[CIT0054] Vanjildorj E Bae TW Riu KZ Yun PY Park SY Lee CH Kim SY Lee HY 2006 Transgenic *Agrostis mongolica* Roshev. with enhanced tolerance to drought and heat stresses obtained from *Agrobacterium*-mediated transformation. Plant Cell, Tissue and Organ Culture 87, 109–120.

[CIT0055] Walter M Chaban C Schütze K 2004 Visualization of protein interactions in living plant cells using bimolecular fluorescence complementation. The Plant Journal 40, 428–438. 15469500 10.1111/j.1365-313X.2004.02219.x

[CIT0056] Wang J Sun PP Chen CL Wang Y Fu XZ Liu JH 2011 An arginine decarboxylase gene PtADC from *Poncirus trifoliata* confers abiotic stress tolerance and promotes primary root growth in *Arabidopsis* . Journal of Experimental Botany 62, 2899–2914. 21282323 10.1093/jxb/erq463

[CIT0057] Wen XP Ban Y Inoue H Matsuda N Moriguchi T 2010 Spermidine levels are implicated in heavy metal tolerance in a spermidine synthase overexpressing transgenic European pear by exerting antioxidant activities. Transgenic Research 19, 91–103. 19544002 10.1007/s11248-009-9296-6

[CIT0058] Xian LH Sun PP Wu J Hu SS Liu JH 2014 Molecular cloning and characterization of *CrNCED1*, a gene encoding 9*-cis*-epoxicarotenoid dioxygenase in *Citrus reshni*, with functions in tolerance to multiple abiotic stresses. Planta 239, 61–77. 24068300 10.1007/s00425-013-1963-4

[CIT0059] Xie C Zhang R Qu Y Miao Z Zhang Y Shen X Wang T Dong J 2012 Overexpression of *MtCAS31* enhances drought tolerance in transgenic Arabidopsis by reducing stomatal density. New Phytologist 195, 124–135. 22510066 10.1111/j.1469-8137.2012.04136.x

[CIT0060] Yáñez M Cáceres S Orellana S Bastías A Verdugo I Ruiz-Lara S Casaretto JA 2009 An abiotic stress-responsive bZIP transcription factor from wild and cultivated tomatoes regulates stress-related genes. Plant Cell Reports 28, 1497–1507. 19652975 10.1007/s00299-009-0749-4

[CIT0061] Yoshida T Fujita Y Maruyama K Mogami J Todaka D Shinozaki K Yamaguchi-Shinozaki K 2015 Four Arabidopsis AREB/ABF transcription factors function predominantly in gene expression downstream of SnRK2 kinases in abscisic acid signalling in response to osmotic stress. Plant, Cell and Environment 38, 35–49. 10.1111/pce.12351 PMC4302978 24738645

[CIT0062] Yoshida T Fujita Y Sayama H Kidokoro S Maruyama K Mizoi J Shinozaki K Yamaguchi-Shinozaki K 2010 AREB1, AREB2, and ABF3 are master transcription factors that cooperatively regulate ABRE-dependent ABA signaling involved in drought stress tolerance and require ABA for full activation. The Plant Journal 61, 672–685. 19947981 10.1111/j.1365-313X.2009.04092.x

[CIT0063] Zhang X Zhang L Dong F Gao J Galbraith DW Song CP 2001 Hydrogen peroxide is involved in abscisic acid-induced stomatal closure in *Vicia faba* . Plant Physiology 126, 1438–1448. 11500543 10.1104/pp.126.4.1438 PMC117144

