# Supplementary material for: PtrABF of Poncirus trifoliata functions in dehydration tolerance by reducing stomatal density and maintaining reactive oxygen species homeostasis
Source: J Exp Bot. 2015 Jun 25;66(19):5911–27. doi: 10.1093/jxb/erv301 (PMC4566982; doi:10.1093/jxb/erv301)
Supplement: Supplementary Data [file supp_66_19_5911__index.html]

PtrABF of Poncirus trifoliata functions in dehydration tolerance by reducing stomatal density and maintaining reactive oxygen species homeostasis — PtrABF of Poncirus trifoliata functions in dehydration tolerance by reducing stomatal density and maintaining reactive oxygen species homeostasis — Supplementary Data 

# PtrABF of *Poncirus trifoliata* functions in dehydration tolerance by reducing stomatal density and maintaining reactive oxygen species homeostasis

## Supplementary Data

Data files

- Supplementary Data - Supplementary Data
