# Supplementary material for: PtrABF of Poncirus trifoliata functions in dehydration tolerance by reducing stomatal density and maintaining reactive oxygen species homeostasis
Source: J Exp Bot. 2015 Jun 25;66(19):5911–27. doi: 10.1093/jxb/erv301 (PMC4566982; doi:10.1093/jxb/erv301)
Supplement: Supplementary Data [file supp_erv301_jexbot143024_file001.pdf]

PtrABF of Poncirus trifoliata functions in dehydration tolerance by reducing stomatal density and maintaining ROS homeostasis

Qinghua Zhang, Min Wang, Wei Wang, Xingzheng Fu, and Ji-Hong Liu

Supplemental Files

Table S1. Primer sequences used for this study.

Purpose	Primers	Sequences (5'-3')	
		Forward	Reverse (5'-3')
Transgenic identification	NPTII	AGACAATCGGCTGCTCTGAT	TCATTTCGAACCCCAGAGTC
	PtrABF-1	CCGGAAACCTCCTCGGATTCCATTGCC	GGTGCCATGTAGTATGGACTGCCCACA
Gene expression	PtrABF	GTGGACTGGGGAAGGATTT	GTCCCTCCAAACTTCATCG
	PtrSPCH	AAGACAGCAGCTACATCGGC	GCATGAGTGAGCGCAAAACA
	PtrMUTE	CATCGCGTGTTGAGGTTGTG	CTCTTCTGGCAACGGTGGAT
	PtrFAMA	AGCGATGTATCGGCTCATGG	TCAGAATCACGGCCCAACTC
	PtADC	AGTCATTGACATCGGGGGCG	GCTTGAACAACGGCAGAGGC
	PtPOD	AAGTTGAATTGGGGAGGCGT	ATCGTATGCGCGCCTGATAA
	NPTII	AGACAATCGGCTGCTCTGAT	TCATTTCGAACCCCAGAGTC
Microarray data verification	Cit.39178.1.S1_s_at	AAACTAAAGCCACGGCCCAA	GAGCTCCGCTTGCATTCTTC
	Cit.12301.1.S1_at	CCCAAACCCATCTGGTCACA	TGCATCTCCGAGACTTCACC
	Cit.19911.1.S1_s_at	ATGGACGAAGTACGCAAGGT	GCCGTCAGTGTCGATCTCAT
	Cit.17318.1.S1_at	TCGACCCTTCCAGTCCATGA	GCGCGGGCATGATACATTTT
	Cit.21952.1.S1_at	TCCGGAATATGCGATGAGTGG	GCCTTCATTCCACAGTTGCC
	Cit.3096.1.S1_s_at	ATCCTCACTGAAAGGCGGAG	TAGCGTCGATGAGGATGGGA
	Cit.29605.1.S1_at	GTCAAGGAGACACCGAGCAA	AGCCTGTGGCCTTCCAATAC
	Cit.18183.1.S1_at	GCTCCACGCAGACAAAATCG	TCACCTTGCACCTTGGATGT
	Cit.29611.1.S1_at	GGACAGCATTGGAAGGGACT	GTTGCTAAACAACCTCGGGC
	Cit.28635.1.S1_at	AGTGGATGAAGCAGGTGTGT	AAGAAATCTGCTGGTGGCGT
Internal control	Actin	ATTGTAACTGGGATGATA	AGAGGCCTCAGAGAAG
Promoter amplification	pPOD	CCACCATTCAATAATGGCATGTGATT	AGAGAGAAGCACCCAGCTATCTT
	pADC	GGGTTGGTGGGCCTAAAGGC	GTGTCGGGGGAGGGAGTTCAG

Yeast one-hybrid assay	pGADT7-PtrABF	TCCCCCGGG ATGGGATCTCAAATGAACTACAAG	CGCGGATCCAGTGTCCTCCTCAAGCACAGTATT
	pAbAi-pPOD’	TCCCCCGGGGCGGCATTTTTTATTTTT	CCGCTCGAGCATGTCGTCAGATCTTTTA
	pAbAi-pADC’	CCGGAATTCATCATGCCACGACTGATGAGC	CCGCTCGAG CTTCGCCTCAATGAAGGATTTGC
Transient expression assay	pGreenII 0800-LUC-pPOD’	CCGCTCGAGTCCGCGGCATTTTTTATTTT	TCCCCCGGGCATGTCGTCAGATCTTTTA
	pGreenII 0800-LUC-pADC’	CCGCTCGAG CCCTCATGGAACACATGAATG	TCCCCCGGG CCTCAATGAAGGATTTGC
	pGreenII 0029 62-SK-ABF	CGCGGATCC ATGGGATCTCAAATGAACTACAAG	TCCCCCGGG CTCAAGCACAGTATTTTGC
Subcellular localization	PtrABF-sub	GTGAATTCATCGATGGATCC ATGGGATCT CAAATGAACTA	TTTACTCATCTCGAGCCCGGG CCAAGGG CCTGTCAGTGTCC
	PtrICE-sub 1	AGTGAATTCATCGATGGATCC ATGCTTTCT AGACTAAACGG	TTTACTCATCTCGAGCCCGGGCCAAGGG CCTGTCAGTGTCC
Y2H	ICE13C	CGGGGTACCCTTGGTGACCCAACAACTGA	CCG*CTCGAG*ATGCCATGGAAGCCGGCTGAAT
	ABF3C	CGG*GGTACC*ATTGGAGCAAGCTGTTGCCA	CCG*CTCGAG*TACTCTACCAAGGGCCTGTCAGT
BiFC	ICE1-BiFC	CG*GGATCC*ATGCTTTCTAGACTAAACGGTGTG	CC*ATCGAT*CATCATGCCATGGAAGCCG
	ABF- BiFC	CG*GGATCC*ATGGGATCTCAAATGAACTACAAG	CC*ATCGAT*CCAAGGGCCTGTCAGTGT
	bZIP63- BiFC	CG*GGATCC*ATGGAAAAAGTTTTCTCCGACG	CCG*CTCGAG*CTGATCCCCAACGCTTCGAAT

Table S2. Comparison of stomatal development genes between *Poncirus trifoliata* and *Arabidopsis thaliana*.

Gene	Sequence number	Homology with Arabidopsis thaliana counterpart			
		E-value	Genes of Arabidopsis thaliana	Accession nunmber	Size
PtrSPCH	orange1.1g042904m	6.E-63	*AtSPCH*	DQ868373.1	1095
PtrMUTE	orange1.1g013752m	2.E-11	*AtMUTE*	NM_111487.2	823
PtrFAMA	orange1.1g014477m	3E-70	*AtFAMA*	NM_113319.4	1729

Table S3. A list of genes down-regulated in transgenic line #10 overexpressing *PtrABF* in comparison with the wild type, as revealed by microarray analysis. The genes with a fold change ≥ 2.0 ($P \leq 0.05$) are included.

**Probe Set ID**	**Product description**	**Fold change**
Cit.18116.1.S1_at	Hypothetical proteins	-3.23598
Cit.18318.1.S1_at	Tetratricopeptide repeat (TPR)-like superfamily protein	-3.06828
Cit.20851.1.S1_s_at	GA20OX1; 2-oxoglutarate (2OG) and Fe(II)-dependent oxygenase superfamily protein	-3.03745
Cit.21081.1.S1_s_at	CRR3; chlororespiratory reduction 3	-2.92676
Cit.36781.1.S1_s_at	SLY1; F-box family protein	-2.90992
Cit.2122.1.S1_at	PIP1; plasma membrane intrinsic protein 1B	-2.67013
Cit.37161.1.S1_at	MLP-like protein 28	-2.49362
Cit.5796.1.S1_at	MLP165; MLP-like protein 165	-2.45404
Cit.16104.1.S1_at	SULTR4; sulfate transporter 4.1	-2.3991
Cit.18106.1.S1_at	SEN1; Rhodanese/Cell cycle control phosphatase superfamily protein	-2.32084
Cit.5796.1.S1_s_at	Choline monooxygenase, putative (CMO-like)	-2.30716
Cit.30315.1.S1_at	SULTR4; sulfate transporter 4.1	-2.29048
Cit.27993.1.S1_at	Rhomboid-related intramembrane serine protease family protein	-2.28357
Cit.2910.1.S1_at	Rhomboid-related intramembrane serine protease family protein	-2.28295
Cit.8476.1.S1_s_at	Tetratricopeptide repeat (TPR)-like superfamily protein	-2.24811
Cit.14227.1.S1_at	Protein of unknown function (DUF581)	-2.24418
Cit.6961.1.S1_at	Reticuline oxidase-like protein	-2.22411
Cit.2990.1.S1_x_at	LSU2;response to low sulfur 2	-2.22248

Cit.2078.1.S1_at	Glycine-rich RNA-binding protein 7	-2.21509
Cit.25338.1.S1_s_at	Glycine-rich RNA-binding protein 7	-2.19157
Cit.12774.1.S1_s_at	CYP76G1; cytochrome P450	-2.15022
Cit.5183.1.S1_s_at	Leucine-rich repeat protein kinase family protein	-2.13264
Cit.10457.1.S1_s_at	CYP82G1; cytochrome P450	-2.09445
Cit.10281.1.S1_s_at	CIPK6; SOS3-interacting protein 3	-2.0916
Cit.10873.1.S1_s_at	HMT3; homocysteine S-methyltransferase 3	-2.07316
Cit.22431.1.S1_s_at	Protein of unknown function (DUF3464)	-2.04993
Cit.29468.1.S1_s_at	KRP2; KIP-related protein 2	-2.03932
Cit.14652.1.S1_s_at	Hypothetical proteins	-2.02075

Supporting information

Fig. S1. Generation and molecular identification of trifoliate orange transgenic plants overexpressing *PtrABF*. (a) Co-culture of the shoot segments. (b) Regeneration of kanamycin-resistant shoots (shown by closed arrow head) from the cutting ends of shoot stem after culture on the selection medium for 60 d, while the kanamycin-sensitive shoot is shown by open arrow head. (c) A representative rooted plants. (d) Genomic PCR confirmation of the kanamycin-resistant plants using NPTII-specific primers (upper panel) or CaMV 35S-PtrABF primers (lower panel). M, molecular marker; +, plasmid DNA (used as a positive control); -, wild type (WT); the numbers indicate different transgenic lines (lines 8 and 10 are designated as #8 and #10, respectively). (e) qPCR analysis of transcript levels of *PtrABF* in WT, two transgenic lines (#8 and #10).

Fig. S2. Comparison of dehydration tolerance between wild type and transgenic plants containing NPTII gene. (a) Molecular characterization of transgenic plants by genomic PCR and RT-PCR. (a) Identification of *NPTII* gene in the transgenic plants via genomic PCR using primers specific to NPTII gene. M, molecular marker; P, plasmid DNA (used as a positive control); N, wild type (WT); NPT-1 and NPT-2 indicate two transgenic lines. (b) RT-PCR analysis of transcript levels of *NPTII* in WT and two transgenic lines (NPT-1 and NPT-2). Actin was used as an internal control. (c) Phenotypes of leaves wild type (WT) and transgenic lines (NPT-1 and NPT-2) and before and after 90 min of dehydration treatment at ambient environment. (d) Measurement of fresh water loss from the detached leaves of WT and transgenic lines at the end of dehydration. (e). Detection of cell death in wild type (WT) and transgenic lines (NPT-1 and NPT-2) after the dehydration.

Fig. S3. Subcellular localization of PtrABF and PtrICE1. Tobacco leaves were transiently transformed with constructs containing either control (35S::YFP, A) or fusion plasmids PtrABF::YFP (B) and PtrICE1::YFP. Images under blight field (left) and fluorescence (middle) were shown. The merged images are show on the right.

Fig. S4. Analysis of H_2O_2 in guard cells. *In situ* accumulation of H_2O_2 in the guard cells of wild type (WT) and transgenic lines was revealed by histochemical staining assay with H2DCF-DA.

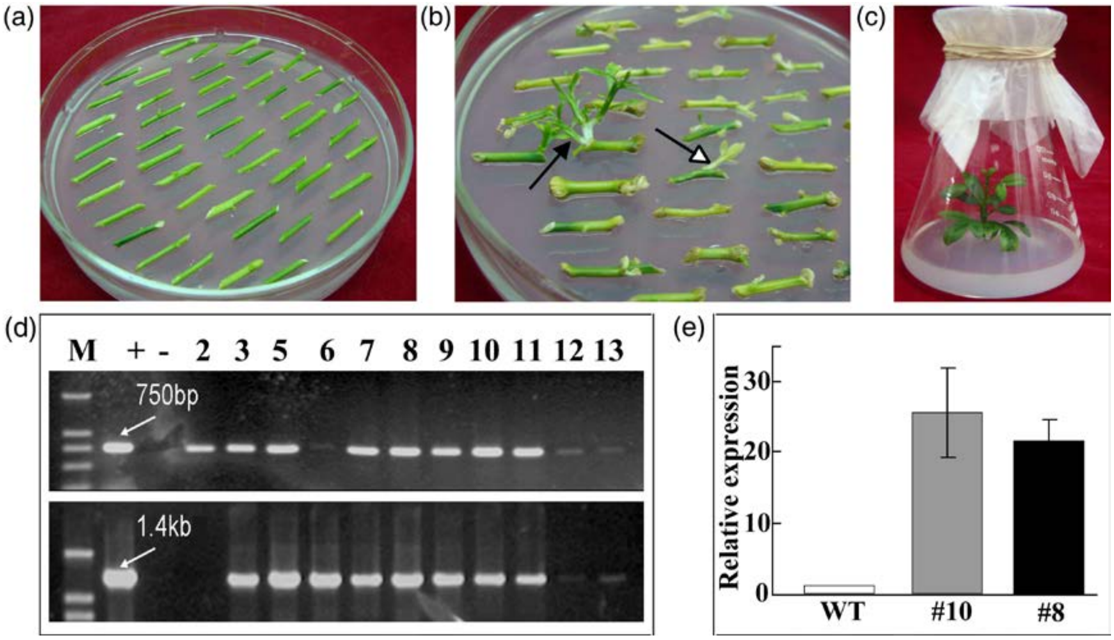
(a)
(b)
(c)
(d)
M + - 2 3 5 6 7 8 9 10 11 12 13
750bp
1.4kb
(e)
Relative expression
30
20
10
0
WT
#10
#8

Fig. S1.

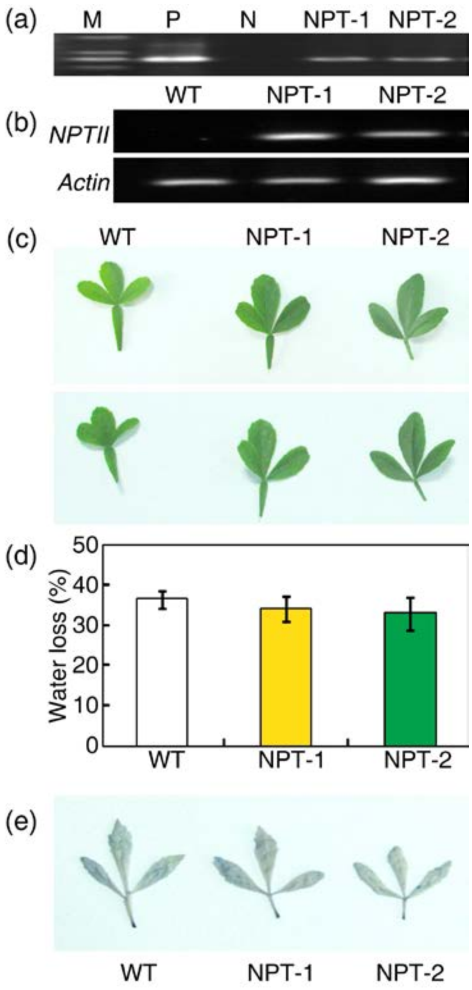
(a)
M
P
N
NPT-1
NPT-2
WT
NPT-1
NPT-2
(b)
NPTII
Actin
(c)
WT
NPT-1
NPT-2
(d)
Water loss (%)
50
40
30
20
10
0
WT
NPT-1
NPT-2
(e)
WT
NPT-1
NPT-2

Fig. S2.

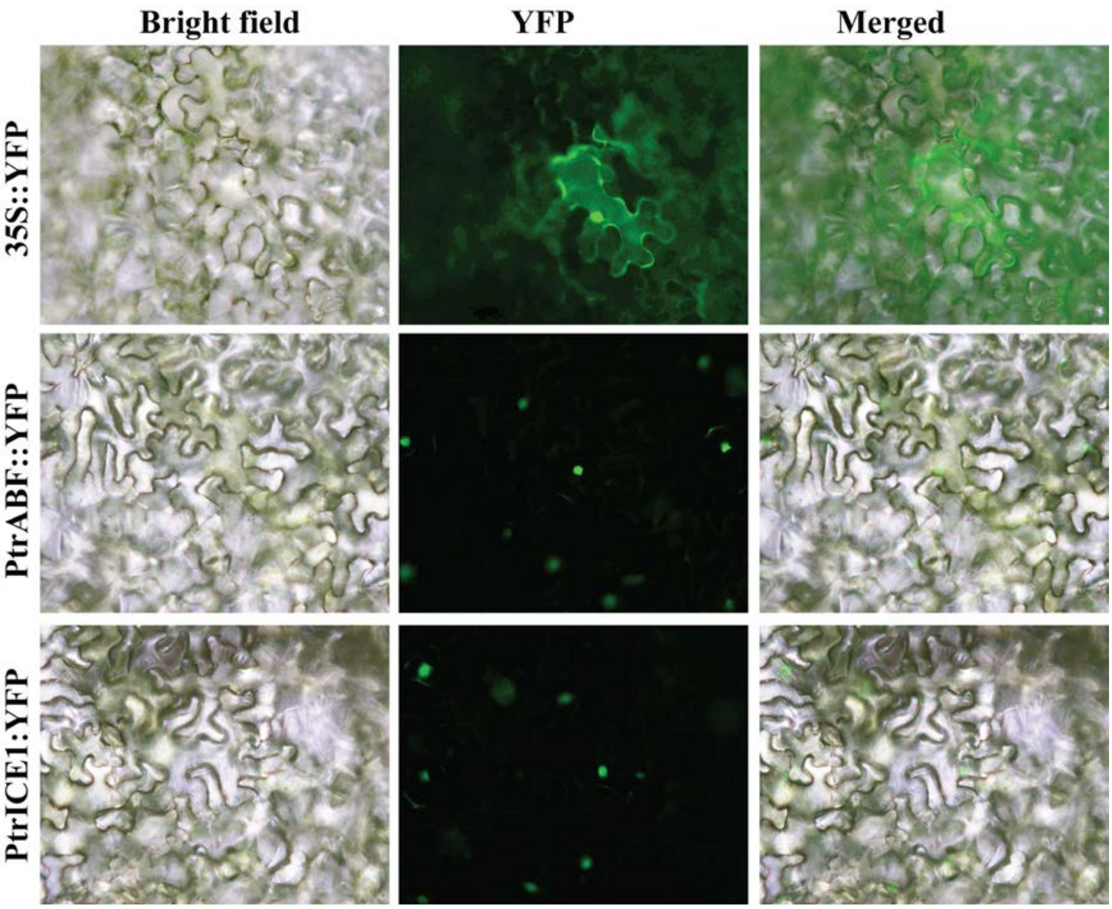

Fig. S3.

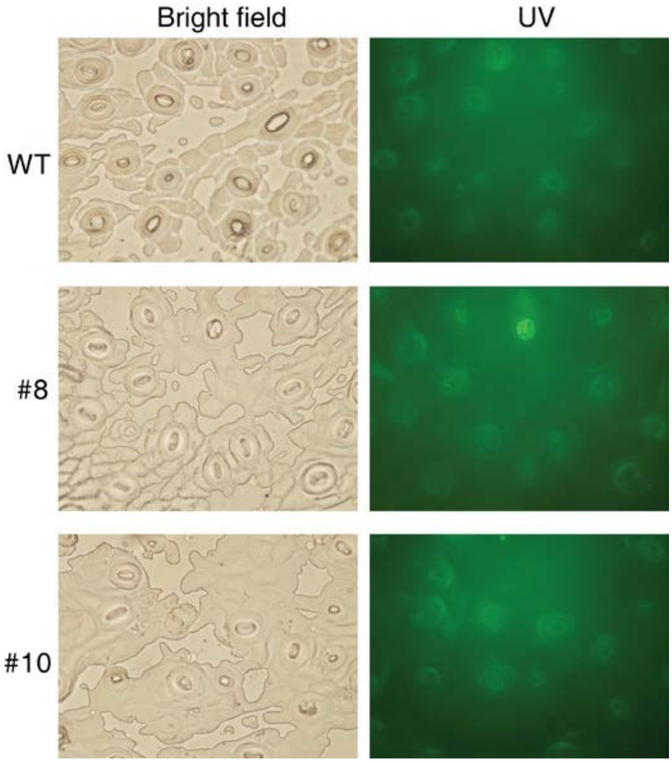

Fig. S4.
